# A mitochondria-targeted molecular phototheranostic platform for NIR-II imaging-guided synergistic photothermal/photodynamic/immune therapy

**DOI:** 10.1186/s12951-022-01679-0

**Published:** 2022-11-11

**Authors:** Sha Yang, Gui-long Wu, Na Li, Minghui Wang, Peixian Wu, Yuxuan He, Wei Zhou, Hao Xiao, Xiaofeng Tan, Li Tang, Qinglai Yang

**Affiliations:** 1 Center for Molecular Imaging Probe, Hunan Province Key Laboratory of Cancer Cellular and Molecular Pathology, Hengyang Medical School, Cancer Research Institute, University of South China, Hengyang, 421001 Hunan China; 2 Cancer Pathology Research Group, Institute of Basic Disease Sciences & Department of Pathology, Xiangnan University, Chenzhou, 423099 Hunan China; 3 Key Laboratory of Tropical Medicinal Plant Chemistry of Ministry of Education, College of Chemistry and Chemical Engineering, Hainan Normal University, Haikou, 571158 Hainan China

**Keywords:** Mitochondria-targeting, NIR-II imaging-guided, Photothermal therapy, Photodynamic therapy, Immunogenic cell death

## Abstract

Phototherapy is a conducive and non-invasive strategy for cancer therapy under light irradiation. Inspiringly, fluorescence imaging in the second near-infrared window (NIR-II, 1000–1700 nm) holds a great promise for imaging-guided phototherapy with deep penetration and high spatiotemporal resolution. However, most phototherapeutics still face great challenges, including complicated synthesis of agents, potential biotoxicity and unsatisfied therapeutic outcomes. Herein, a near-infrared laser triggered molecular photosensitizer FEPT, modified with triphenylphosphine PEGylation (PEG2000-TPP), is developed for NIR-II imaging-guided mitochondria-targeting synergistic photothermal therapy (PTT)/photodynamic therapy (PDT)/immune therapy (IMT). The mitochondria-targeting photosensitizer FEPT can produce reactive oxygen species (ROS) and hyperpyrexia upon 808 nm laser irradiation, resulting in mitochondrial dysfunction and photo-induced apoptosis via caspase-3 pathway. Phototherapy-induced hyperthermia or ROS triggers the release of immunogenic intracellular substrates from dying tumor cells, thereby promoting the activation of antitumor immunity. Herein, this work provides a practicable strategy to develop a molecular phototheranostic platform for imaging-guided cancer therapy via mitochondria-targeting.

**Graphical Abstract:**

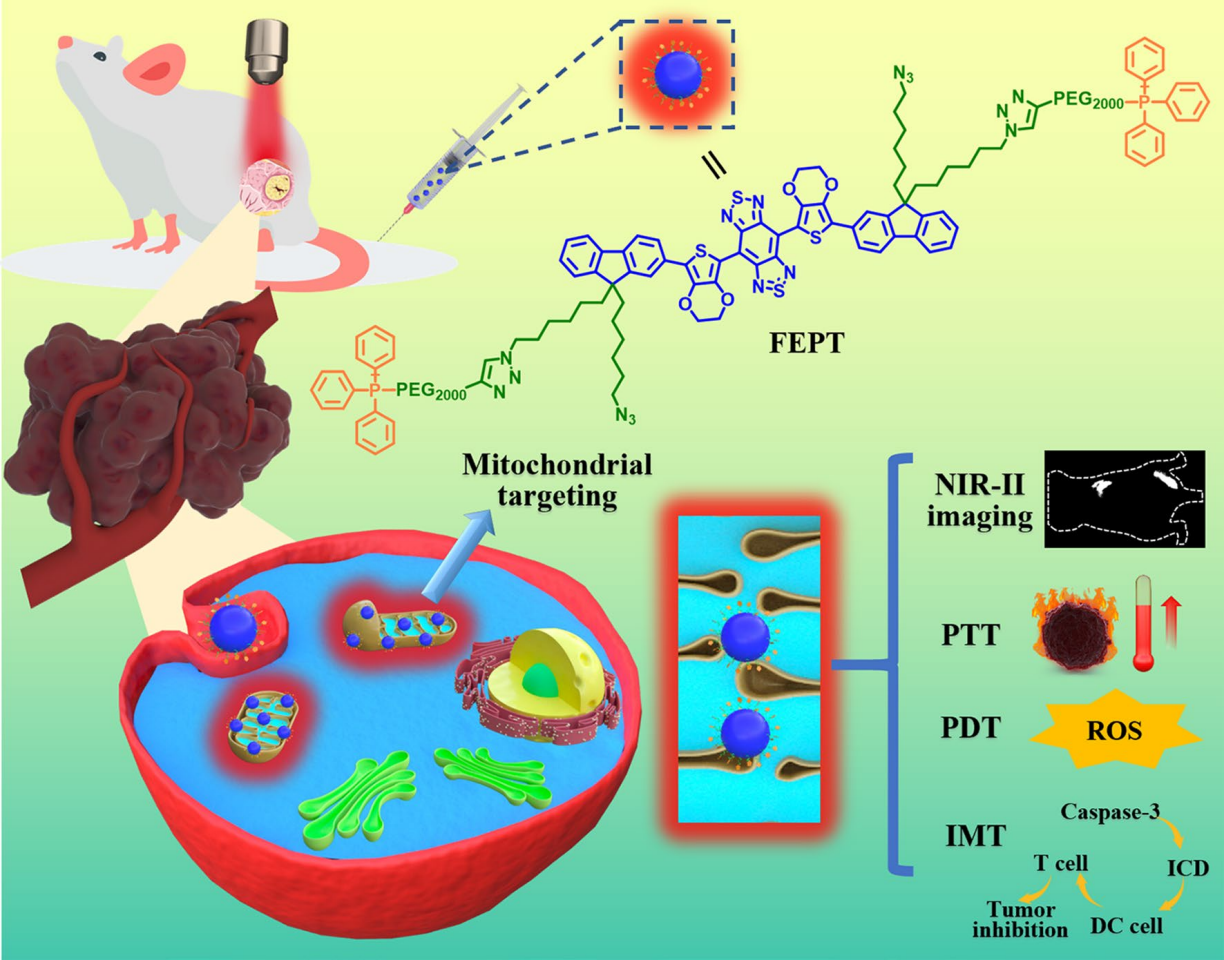

**Supplementary Information:**

The online version contains supplementary material available at 10.1186/s12951-022-01679-0.

## Background

Phototherapy has been recognized as a potential and indispensable tool for cancer therapy [1, 2]. With the merits of high spatiotemporal precision in *in-situ* fluorescence imaging and effective multi-model synergistic therapy, phototheranostics have thrived as a promising platform for cancer therapy. At present, photodynamic therapy (PDT) and photothermal therapy (PTT) have attracted extensive attention due to their advantages of low toxicity, high safety, non-invasive, wide treatment range and low drug resistance [3, 4]. PDT is a locally targeted therapy that uses photosensitizer (PS), light, and oxygen to selectively kill tumors. However, low oxygen levels in hypoxic tumor microenvironments (TME) result in low ROS production and might result in poor therapy for PDT. Unlike PDT, PTT uses an external light source (usually near infrared (NIR) light) and photothermal agents as heat sources to kill cancer cells through thermal damage (converting light energy to heat) without any additional mitochondrial oxygen for promising cell killing [5, 6]. Upon NIR laser irradiation, the combination of these two techniques is identified as a path-breaking strategy to overcome respective shortcomings and acquire synergistic effects with outstanding treatment outcomes [7, 8]. Nevertheless, the therapeutic effect of the most reported phototherapeutic agents is still limited by multiple concerns, including complicated synthesis of agents, poor biocompatibility, low NIR absorption capability and photothermal conversion efficiency (PCE) [9, 10]. Notably, photosensitizers delivered in cellular crucial positions, such as organelle, can effectively enhance anticancer therapeutic effect [11].

Mitochondria, as crucial organelles, regulate many significant cellular behaviors, such as cellular signaling, growth, differentiation, metabolism, apoptosis and death [4]. In order to improve the efficiency of cancer therapy, PDT, PTT, and related combination therapies targeting mitochondria have attracted wide attention [4]. Remarkably, the mitochondrial membrane potential (ΔΨ) with − 220 mV in cancer cells are more hyperpolarized than that (− 140 mV) in normal cells, making the uptake of lipophilic cationic compounds be raised by 100–1000 folds [4]. According to the specific ΔΨ characteristic of mitochondrion, a large amount of mitochondria-targeted therapeutic agents have been rapidly developed, including triphenyl phosphonium (TPP), rhodamine, F16, dequalinium (DQA), and guanidine [12, 13]. Up to now, TPP is the most widely used and valid for targeting mitochondria owing to its simple purification and modification [14]. Moreover, TPP targeting mitochondria for cancer therapy is based on two points: (i) mitochondrial membrane is particularly sensitive to heat, and (ii) mitochondrial membrane becomes permeable easily [15]. Therefore, a favorable mitochondria-targeting PS should take account of PDT and PTT to increase the therapeutic effect as well as reduce the therapeutic dose for cancer therapy. With the growing development of phototheranostics, phototheranotics-guided cancer therapies become more and more promising and significant . Since mitochondria provide energy for cancer cell growth, targeting mitochondria for cancer therapies can directly inhibit mitochondria from providing energy to cancer cells, resulting in cells death [16, 17]. However, the emit lights in the most of the current mitochondria-targeted therapy probes are in the visible (400-750 nm) or the first near-infrared (NIR-I,750-1000 nm) region [12, 18, 19]. Inspiringly, in recent years, fluorescence imaging in the second near-infrared window (NIR-II, 1000–1700 nm) holds a great promise for imaging-guided phototherapy due to its deep penetration and high spatiotemporal resolution [20, 21]. Therefore, developing multi-functional phototherapnostic agents for NIR-II imaging-guided mitochondria-targeting phototherapy is rewarding for cancer therapy.

**Scheme 1:**
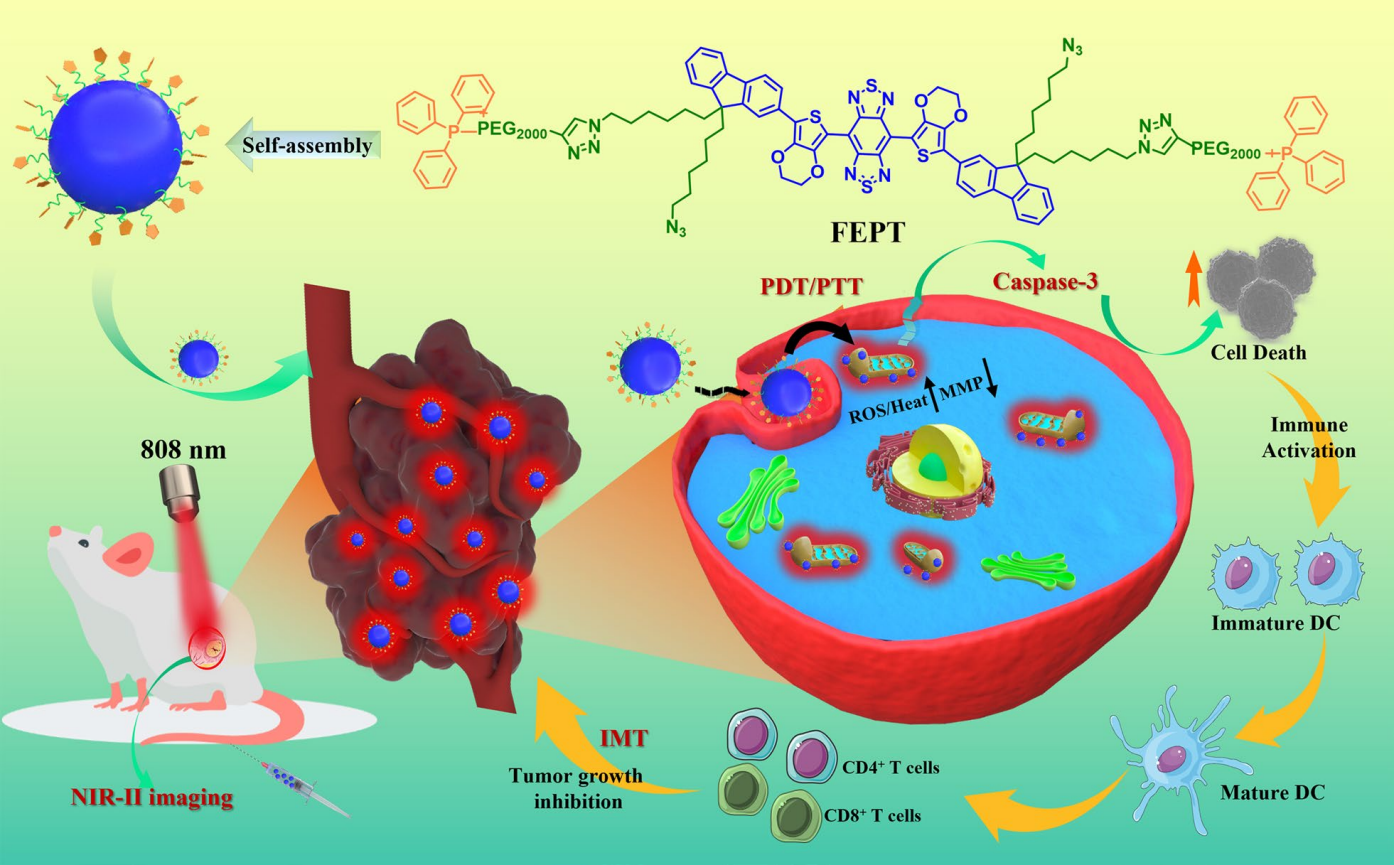
Schematic illustration of molecular photosensitizer FEPT for NIR-II imaging-guided mitochondria-targeting synergistic PTT/PDT/IMT. *PTT* photothermal therapy, *PDT* photodynamic therapy, *IMT* immune therapy, *DC* dendritic cells

In our previous study, the outstanding performance fluorophore IR-FE has high QY of 31% in toluene , representing an excellent organic dye in the reported NIR-II region so far [22]. Based on the IR-FE,  after the modification of triphenylphosphine PEGylation (PEG2000-TPP), a mitochondria-targeting organic photosensitizer FEPT was successfully synthesized for NIR-II imaging-guided mitochondria-targeting synergistic PTT/PDT/IMT with a single 808 nm laser irradiation (Scheme 1). The ingenious design of FEPT should fulfill two critical requirements to enhance the efficiency of PTT/PDT/IMT for cancer therapy: (i) efficient light absorption with not high fluorescence QY, to maximize the conversion of absorbed photoenergy into thermal energy and (ii) precisely mitochondrial localization of photosensitizers to enhance the expected phototoxicity [23]. With the excitation of 808 nm laser, FEPT could obtain obvious NIR-II fluorescence signals (QY:1.64% in water), contributing to in vivo NIR-II imaging with a real-time cancer location and monitoring the therapeutic process. Moreover, FEPT also presented a superb photothermal conversion efficiency (56.8%) and produced a large amount of ROS under 808 nm laser irradiation, which could easily cause mitochondrial dysfunction in vivo, eventually leading to cancer cells death. By mitochondria-targeting, FEPT can maximize the synergistic effect of PTT and PDT, and then induce powerful ICD, as well as activate the specific cancer immune response in vivo, effectively inhibiting the growth of tumors. This work presents a practicable strategy to develop a molecular phototheranostic platform for imaging-guided synergistic PTT/PDT/IMT via mitochondria-targeting.

## Results and discussion

### Preparation and characterization

The organic fluorophore IR-FE, constructed with benzobisthiadiazole (BBTD) as the electron acceptor (A), 3,4-ethylenedioxy thiophene (EDOT) as the electron donor (D) and fluorene as the shielding units (S), exhibits fluorescence peaked at 1013 nm with a QY of 31% in toluene [22, 24]. In order to synthesize the mitochondria-targeting photosensitizer FEPT, all bromide in IR-FE was substituted by the azide group, and then the triphenylphosphine and alkyne-terminated polyethylene glycol (Alkyne-PEG2000-TPP) were click reacted with the former trinitride to afford FEPT for mitochondrial-targeting. At the same time, the alkyne-PEG2000 was reacted with the former trinitride to obtain FEP as a control. The relative reaction formula of FEP and FEPT were shown in Additional file 1: Fig. S1. The specific synthesis and characterization of IR-FE were shown in Additional file 1: FigS. S2–S9 at length. FEP and FEPT were characterized with Nuclear magnetic resonance (NMR) and High-Resolution Mass Spectrometry (HRMS) (Additional file 1: Fig. S10–16).

**Fig. 1:**
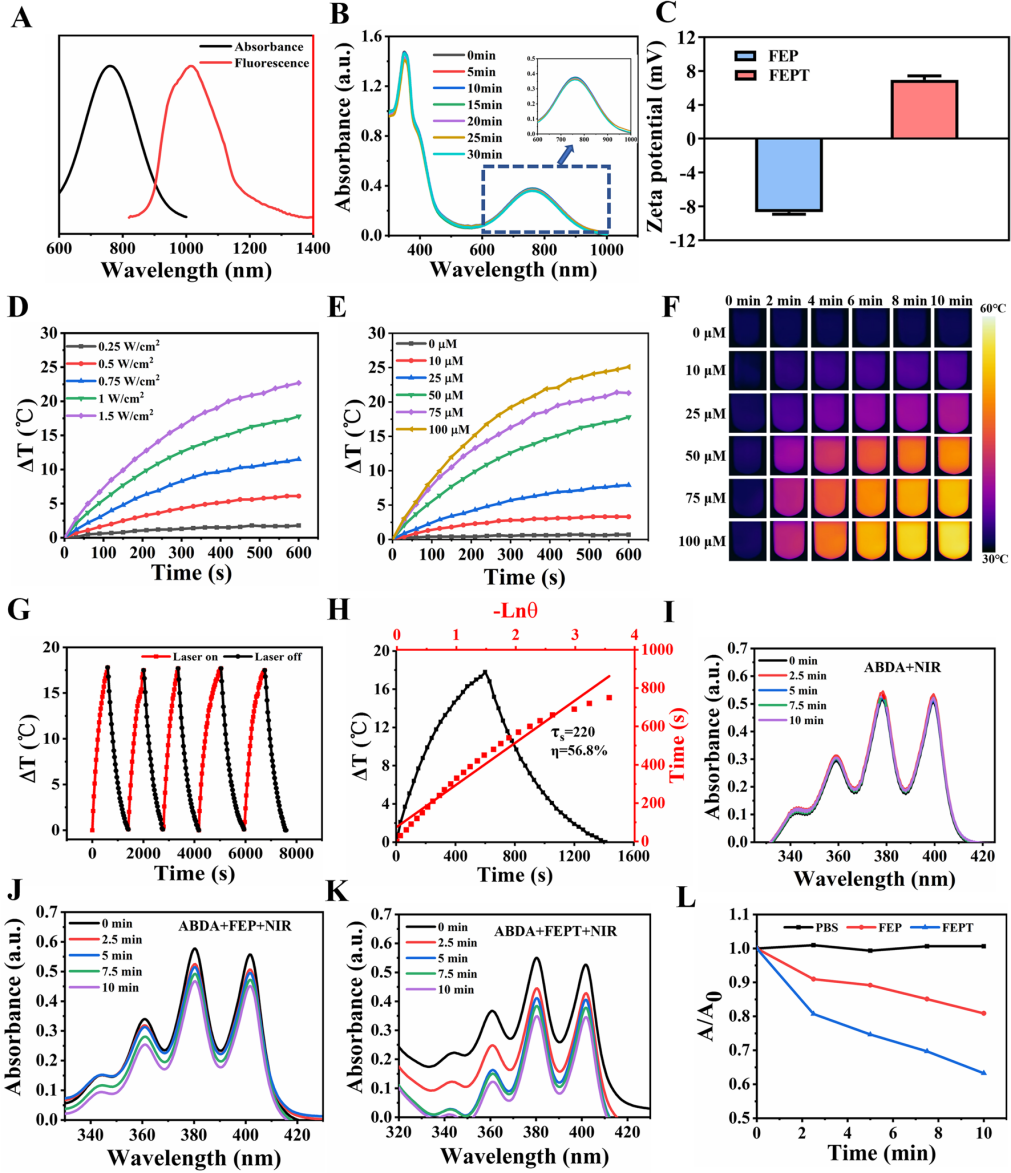
**A** NIR absorption (black line) and NIR-II fluorescence emission spectra of FEPT (red line) (solvents: water). **B** The absorption spectra of FEPT after different times of laser irradiation (808 nm, 1.0 W/cm^2^). **C** Zeta potentials of FEP and FEPT in water. Heating curves of FEPT upon 808 nm laser irradiation with **D** various powers and **E** different concentrations upon 808 nm laser irradiation. **F** IR thermal images of heating process of various concentrations of FEPT upon 808 nm laser irradiation. **G** Photothermal stability of FEPT (50 µM). Curves showing the temperature change of FEPT with five ON/OFF cycles involving irradiation with an 808 nm laser (1.0 W/cm^2^) for more than one hour followed by passive cooling.** H** Photothermal conversion performance curves of FEPT. **I-K** Measurement of ^1^O_2_ production in 4T1 cells after pre-incubation with FEP or FEPT under 808 nm laser irradiation. **L** Plots of relative absorbance of ABDA of different groups in PBS. A_0_ and A are absorbances of ABDA at 380 nm before and after 808 nm laser irradiation for ^1^O_2_, respectively

The absorption of FEPT  showed significant signals around 405 nm and 808 nm (Additional file1: Fig.S17A), and the NIR-II fluorescence emission spectra of FEP and FEPT in water were determined under the 808 nm laser excitation (Fig. 1A and Additional file 1: Fig. S17B). Particularly, the absorption of FEPT was stable after the irradiation with different times under 808 nm laser (1.0 W/cm^2^) (Fig. 1B). In addition, the QY of FEPT in water was 1.64% under 808 nm laser excitation using IR-FEP (IR-FE modified with PEG600) dye as a reference [24]. These results indicated that the FEPT can afford a steady NIR-II imaging. While, with the 405 nm laser excitation, the fluorescence emission of FEP and FEPT were both in the range of 450 ~ 700 nm (Additional file 1: Fig. S17C), which make them apply for cell imaging with a confocal laser microscope system (CLSM). Importantly, compared to the FEP with a negative charge of − 8.67 ± 0.33 mA, the zeta potential of FEPT was with a positive charge of 6.97 ± 0.64 mA (Fig. 1C), significantly contributing to targeting mitochondria for cancer therapy via the electric charge effect.

Thereafter, the photothermal performance of probes was evaluated. The temperature rising curves of FEPT(50 µM) with varying laser power densities (0.25, 0.5, 0.75, 1, 1.25, and 1.5 W/cm^2^) (Fig. 1D), and with various concentrations (0, 10, 25, 50, 75, and 100 µM) were recorded under 808 nm laser irradiation (1.0 W/cm^2^, 10 min) (Fig. 1E). Rapid photothermal effects occurred on the laser irradiation at the dose of 50 µM in the FEPT solution. The related infrared (IR) thermal images were shown in Fig. 1F. The photothermal stability of FEPT (50 µM) in water was further assessed by five heating/cooling cycles (Fig. 1G). According to the reported methods [25, 26], no obvious decline of temperature was observed and showed a much more outstanding photothermal conversion performance of 56.8% than that of FEP (40%) (Fig. 1H, Additional file 1: Fig. S17D) and other existing photothermal agents [25, 27, 28]. In order to evaluate the in vivo stability of FEPT, the absorbance and NIR-II fluorescence intensity in different PH solutions were measured (Additional file 1: Fig. S18A, B), and these results showed that there is no drop of absorbance or NIR-II fluorescence in FEPT solutions with different PH , indicating that FEPT was free from the influence of the physiological environment in vivo. Additionally, as an amphiphilic fluorescent probe, FEPT could form nanoparticles via self-assembly. size and morphology of the FEPT were characterized by dynamic light scattering (DLS) and transmission electron microscopy (TEM), respectively. The results showed that thesizes of the FEPT measured by DLS and TEM were 58 nm and 55 nm (Additional file 1: Fig. S18C, D), respectively. Consequently,the good stability and excellent photothermal performance of FEPT make it a favorable phototheranostics agent for NIR-II fluorescence image-guided photothermal therapy in vivo. To evaluate ROS production of FEPT, 9,10-Anthracenediyl-bis (methylene) dimalonic Acid (ABDA), a singlet oxygen (^1^O_2_) probe, was ultilized to measure ^1^O_2_ under 808 nm laser irradiation (Fig 1I-L). The results indicated that the FEPT group could produce more ^1^O_2_ than the FEP and PBS groups under 808 nm laser irradiation, demonstrating that FEPT was a candidate photosensitizer for PDT in cancer therapy.

### Cellular uptake and mitochondria-targeting imaging

**Fig. 2:**
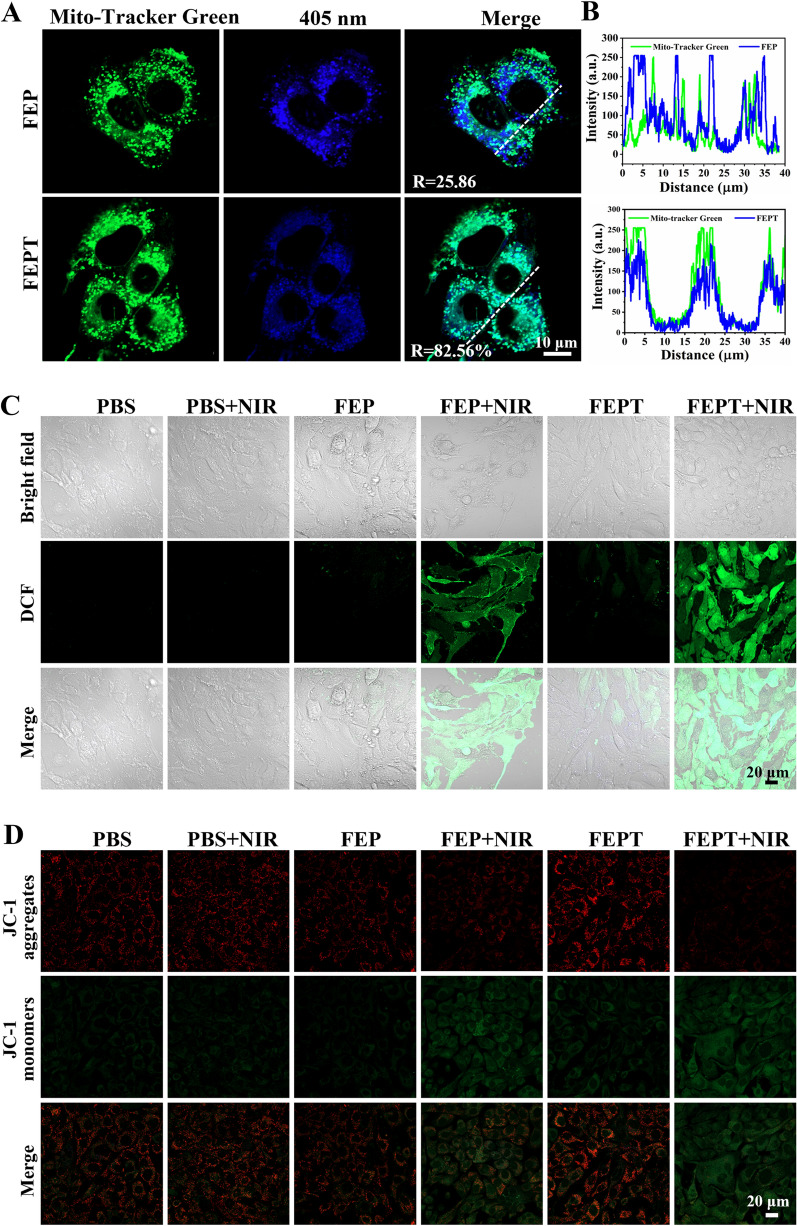
**A** Confocal microscopy images of 4T1 cells after incubation with MTG and FEP or FEPT. Scale bar: 10 µm. **B** Intensity profiles of linear regions of interest between FEP or FEPT and MTG. Fluorescence imaging of **C** intracellular ROS burst production, and **D** JC-1 stained cells under different conditions. The cells were co-stained with DCFH-DA (10 µM) or JC-1 (2 µM). Scale bar: 20 μm

The cellular uptake behavior of FEPT in 4T1 cells was primarily investigated by CLSM (Additional file 1: Fig S19). Strong fluorescence signals in 4T1 cellswere observed at specific time points (0, 2, 4, and 6 h), suggesting that FEPT could be effectively internalized by cancer cells. In order to obtain the specificity to mitochondria, mitochondria-targeted ligand triphenylphosphine (TPP) was introduced. To further assess the targeting ability of FEPT to mitochondria, 4T1 cells were incubated with FEPT for 4 h and then stained with Mito-tracker Green (MTG), which was employed to label mitochondria before the imaging experiment. The overlapping degree of targeted FEPT and MTG was quantitatively evaluated using Pearson’s correlation coefficients (PCCs), and the ability to target mitochondria was evaluated with CLSM in 4T1 cells. As shown in Fig. 2A, B, the intrinsic fluorescence of FEPT (blue) was observed to be completely overlapped with MTG (green) (PCC: 83.08%), implying its good targeting ability to mitochondria. In contrast, the PCC of the non-targeted FEP was only 25.86%, confirming that the mitochondria-targeting FEPT could effectively accumulate in the mitochondrial sites. Considering the outstanding optical properties, positive charge, and precise mitochondria-targeting ability, FEPT was superior to non-targeted FEP probe and was potential for cancer therapy in vitro and in vivo*.*

### Intracellular ROS generation and mitochondrial membrane potential detection

Studies have shown that ROS can damage mitochondria of cancer cells, inducing cells to death [29, 30]. Upon 808 nm laser irradiation, the cellular ROS generation of FEP or FEPT in 4T1 cells was measured by a ROS probe, 2,7-Dichlorodi-hydrofluorescein diacetate (DCFH-DA), which would convert dichlorodihydrofluorescein (DCFH) to 2′,7′-dichlorofluorescein (DCF) with the presence of ROS [31]. After the incubation of  PBS, FEP or FEPT in 4T1 cells for 4 h, DCFH-DA was added and then, the irradiated groups were exposed to 808 nm laser (1.0 W/cm^2^, 10 min). The groups of PBS, FEP or FEPT without laser irradiation showed negligible green fluorescence. However, after the 808 nm laser irradiation, there was a significant green fluorescence inside the cells treated with FEPT, higher than that treated with FEP and PBS (Fig. 2C, Additional file 1: Fig S20A), suggesting that FEPT could generate ROS in the complexity of biological systems upon 808 nm laser irradiation and was potential for PDT in vivo.

ROS and heat can easily damage mitochondria with a significantly changed ΔΨm [15, 32]. ΔΨm can reflect the different cellular status and could be used for evaluating the therapeutic effects [20, 33]. JC-1, as a typical mitochondrial probe, was usually utilized to assess the state of mitochondria. In the normal mitochondria, JC-1 exists in the aggregated state with red fluorescence (580 nm) for its high membrane potential, and in the destructed mitochondria, JC-1 exists in the monomeric state with green fluorescence (530 nm) for the low membrane potential. Hence, the status of mitochondria and cells is assessed with the ratio of red/green fluorescence, and the changes in ΔΨm of mitochondria were determined. As shown in Fig. 2D and Additional file 1: Fig. S20B, the cells in PBS-treated , unirradiated FEP and FEPT groups that stained with JC-1 exhibited a high-level of red fluorescence and relatively weak green fluorescence, indicating the decreased ΔΨm (% of control) of those groups was nearly 100%. In comparison, the irradiated FEPT group displayed the brightest green fluorescence and extremely weak red fluorescence under 808 nm laser irradiation, resulting in relatively low decreased ΔΨm of 13.8%, which is lower than that of the irradiated FEP group (24.65%). All this results indicated that FEPT could not only facilitate the mitochondria-targeting but also induce the mitochondria-mediated cell apoptosis with NIR irradiation.

### Cellular phototherapy

**Fig. 3:**
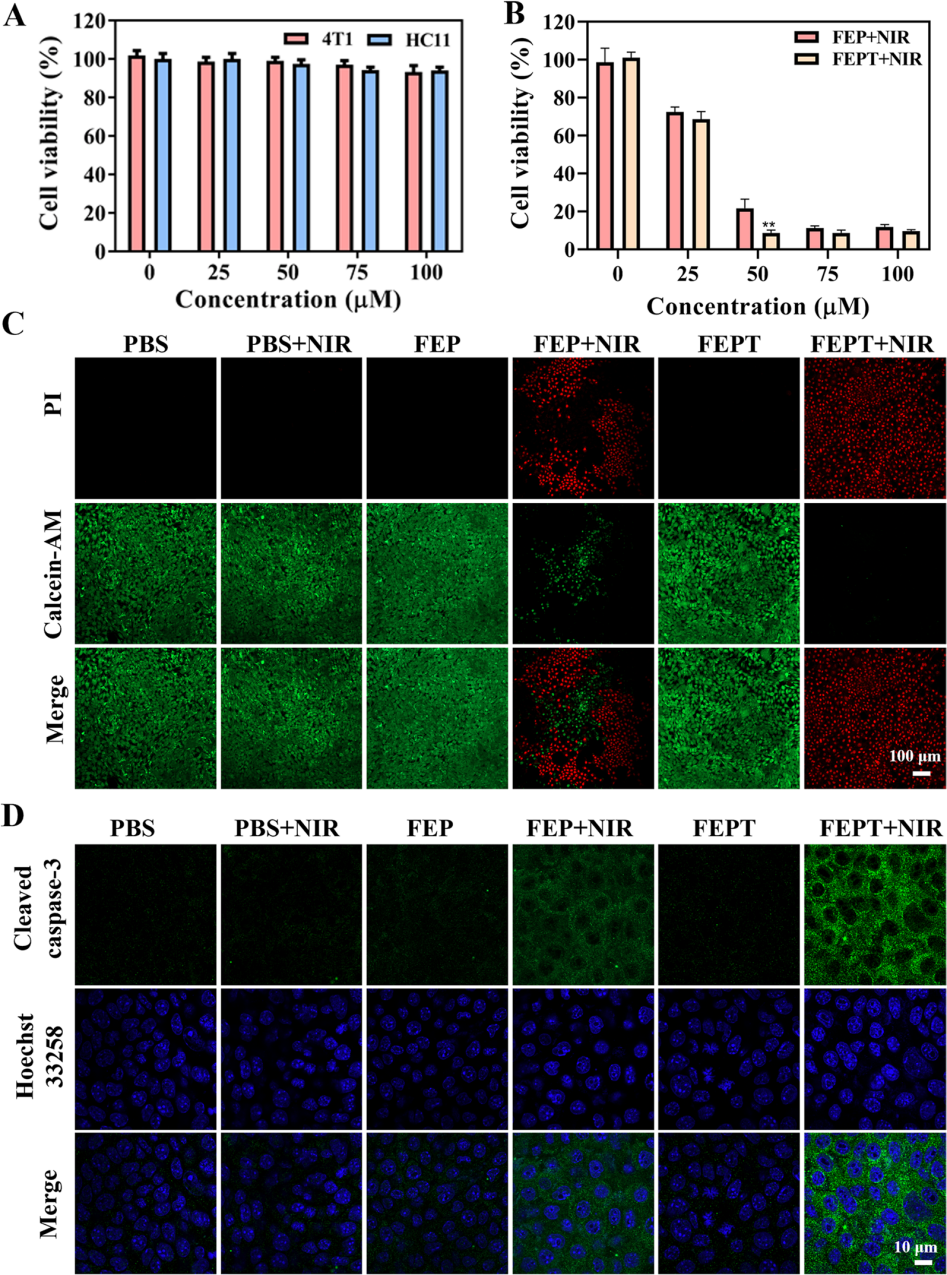
**A** In vitro cell viability of 4T1 and HC11 cells treated with various concentration of FEP or FEPT in the dark. **B** Cell viability of 4T1 cells treated with FEP or FEPT with irradiation (808 nm, 1.0 W/cm^2^). **C** Live/dead staining of FEP or FEPT treated 4T1 cells with 808 nm laser irradiation (1.0 W/cm^2^) for 10 min. The live cells were stained by Calcein-AM (green), whereas dead cells were stained by PI (red). Scale bar: 100 μm. **D** Cleaved caspase-3 staining of FEP or FEPT treated 4T1 cells with 808 nm laser irradiation (1.0 W/cm^2^) for 10 min by CLSM. The primary antibody is cleaved caspase-3 polyclonal antibody, while the second antibody is Goat Anti-Rabbit IgG-Alexa Fluor 488. Scale bar: 10 μm. Error bars: mean ± SD (n = 3). *P < 0.05; **P < 0.01; ***P < 0.001

To evaluate the cytotoxicity of FEPT in vitro, the cell viabilities were investigated by Cell Counting Kit-8 (CCK8) assays in the 4T1 (mouse mammary carcinoma cell) and HC11 (the mouse mammary epithelial cell) cells with different concentrations of FEPT in the dark. No obvious cytotoxicity of FEPT was observed even at a higher concentration up to 100 µM, suggesting that FEPT had good biocompatibility toward 4T1 and HC11 cells (Fig. 3A). Subsequently, the photothermal ablation of 4T1 cells induced by FEP or FEPT was conducted under 808 nm laser irradiation (1.0 W/cm^2^, 10 min). In comparison to the unirradiated FEPT and PBS-treated groups, both FEP and FEPT could effectively induce 4T1 cells to death with 808 nm laser irradiation. Nevertheless, the cell viability in the irradiated FEPT group was less than 10%, much lower than that in irradiated FEP group of 21.6% (Fig. 3B). Based on the CCK8 experiment, to observe the toxicity of probes intuitively, live/dead cell staining assays were carried out by incubating FEP or FEPT.After 808 nm laser, irradiation Calcein Acetoxymethyl Ester (Calcein AM, green for live cells) and propidium iodide (PI, red for dead cells)  were stained. As shown in Fig. 3C, negligible red emission but bright green fluorescence was detected in 4T1 cells treated with FEPT without irradiation, presenting good biocompatibility and low toxicity of FEPT in the dark. Upon 808 nm laser irradiation for 10 min (1.0 W/cm^2^), a large amount of dead cells in FEPT-treated groups were significantly increased, higher than that in FEP-treated group, indicating that the cancer cells can be effectively killed by FEPT with 808 nm laser irradiation. All these results demonstrated that the mitochondria-targeted molecular phototheranostic agent FEPT exhibited effective PDT and PTT for killing cells in vitro under NIR irradiation.

### Cellular apoptosis pathway in cellular phototherapy

The activation of caspase-3 is a significant indicator in stress-mediated apoptotic pathways [34, 35]. Immunofluorescence (IF) staining method was utilized to investigate the caspase-3 activation in 4T1 cells with different treatments. After incubation with PBS,FEP or FEPT for 4 h in 4T1 cells, those irradiated groups were exposed to 808 nm laser for 10 min (1.0 W/cm^2^). The fluorescence intensities of the unirradiated FEP and FEPT groups were similar with the PBS-treated groups, revealing weak activity of caspase-3 (Fig. 3D and Additional file 1: Fig. S20C). Whereas, the cleaved caspase-3 was increased in the irradiated FEPT group. Notably, the increased caspase-3 activity in the FEPT-treated group was much greater than that in the FEP group after 808 nm laser irradiation. Similar results were obtained from the detection of cleaved caspase-3 with different treatment in 4T1 cells by Enzyme-Linked Immunosorbent Assay(ELISA), showing that the expression of cleaved caspase-3 in the irradiated FEPT group was the higher than other groups, including the irradiated FEP group (Additional file 1: Fig S20D). Based on the experiments mentioned above, the mitochondria-targeting photosensitizer FEPT could effectively produce abundant ROS and hyperpyrexia, finally inducing cell apoptosis via the caspase-3 pathway.

### In vivo NIR-II fluorescence imaging

**Fig. 4:**
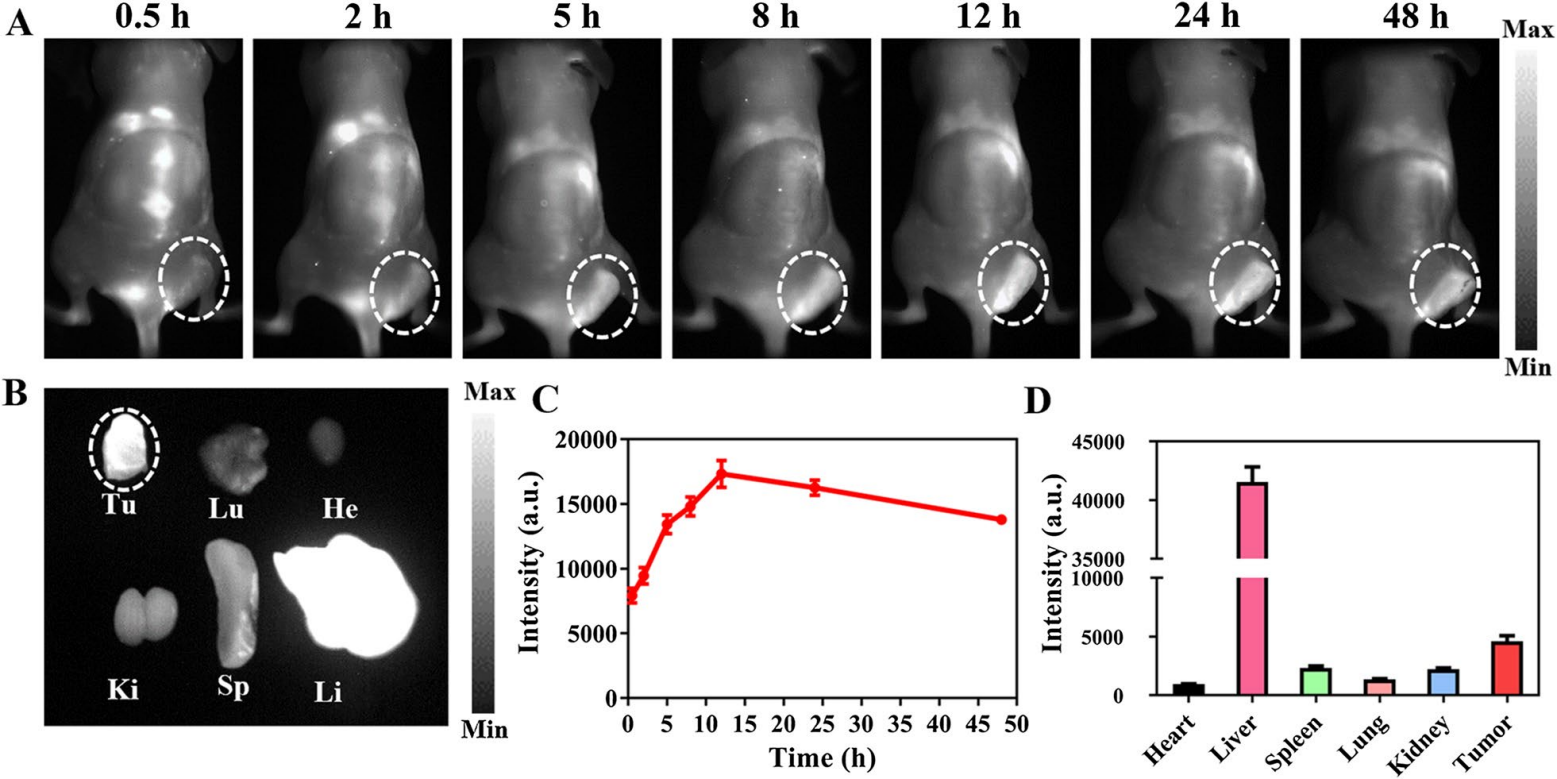
Fluorescence investigation on the distribution patterns of FEPT in 4T1 tumor-bearing Balb/c mice. **A** In vivo NIR-II fluorescence imaging of 4T1 tumor-bearing mice after intravenous injection of FEPT at 0.5, 2, 5, 8, 12, 24, and 48 h, respectively. Tumors are highlighted in dotted circles. **B** Fluorescence images of sacrificed major organs and tumors at 48 h post-injection (under 808 nm laser excitation, emission filters: 910Lp + 1100Lp, exposure time: 150 ms). The NIR-II imaging curves of **C** tumors and **D** total radiation efficiency of FEPT in major organs and tumors at 48 h post-injection. Tu: tumor, Lu: lung, He: heart, Ki: kidney, Sp: spleen, Li: liver. Data are presented as Mean ± SD (n = 3)

The NIR molecular fluorescence IR-FE has been applied for in vivo NIR-II imaging [22]. Herein, the real-time fluorescence imaging of FEPT (100 μl, 200 μM) was captured with NIR-II image system to explore in vivo NIR-II fluorescence imaging after intravenous injection of FEPT into 4T1 tumor-bearing Balb/c mice. In vivo fluorescence images were monitored at predetermined times (0.5, 2, 5, 8, 12, 24, and 48 h). During the first half hour, the fluorescence signal of FEPT was began to accumulate in the tumor sites. Then, the fluorescence intensity in the tumor sites gradually increased over time until peaking at 12 h post-injection. Subsequently, the fluorescence signals in the tumor sites were gradually decreased along with time (Fig.4A and C. The mice were sacrificed at 48 h post-injection, and the excised major organs and tumors were harvested and imaged (Fig. 4B). A maximum accumulation of FEPT in the tumor followed by the liver, and other major organs were with little NIR fluorescence (Fig. 4D). As we all know, positively charged and large-sized molecules tend to accumulate in livers, and possibly be cleared from the hepatobiliary pathway [36]. Herein, FEPT might be cleared from the body by hepatobiliary pathway. These results confirmed that the mitochondria-targeting FEPT probe would provide a tumor accumulation platform for following PDT /PTT in vivo.

### In vivo cancer phototherapy

To assess the biotoxicity in vivo, FEP, FEPT (100 μl, 500 μM) and PBS (100 μl) were intravenously injected into Balb/c female mice (n = 3 per group, ~ 18 g each), respectively. Hematoxylin–eosin (H&E) staining images showed that the morphology of major organs (heart, liver, spleen, lung, and kidney) in different-treated mice had no evident difference (Additional file 1: Fig. S21). Blood routine examination was also performed at 15 days post-injection. Results indicated that no significant changes in blood routine indexes of those three groups, indicating that FEPT have a good biocompatibility for application in vivo (Additional file 1: Fig. S22A). In addition, the biochemistry indexes, including alanine transaminase (ALT), aspartate aminotransferase (AST), alkaline phosphatase (ALP), γ-glutamyltranspeptidase (γ-GT), creatinine (CREA), urea (UREA) and uric acid (UA) were also detected (Additional file 1: Fig. S22B). The biochemistry indexes in the FEPT group were at the normal level, which were similar to that in the FEP and PBS groups, indicating that these probes have no obvious toxicity to the kidneys and livers. All these results effectively confirmed that FEPT is a hopeful and potential NIR-II fluorophore for clinical application in cancer therapy.

**Fig. 5:**
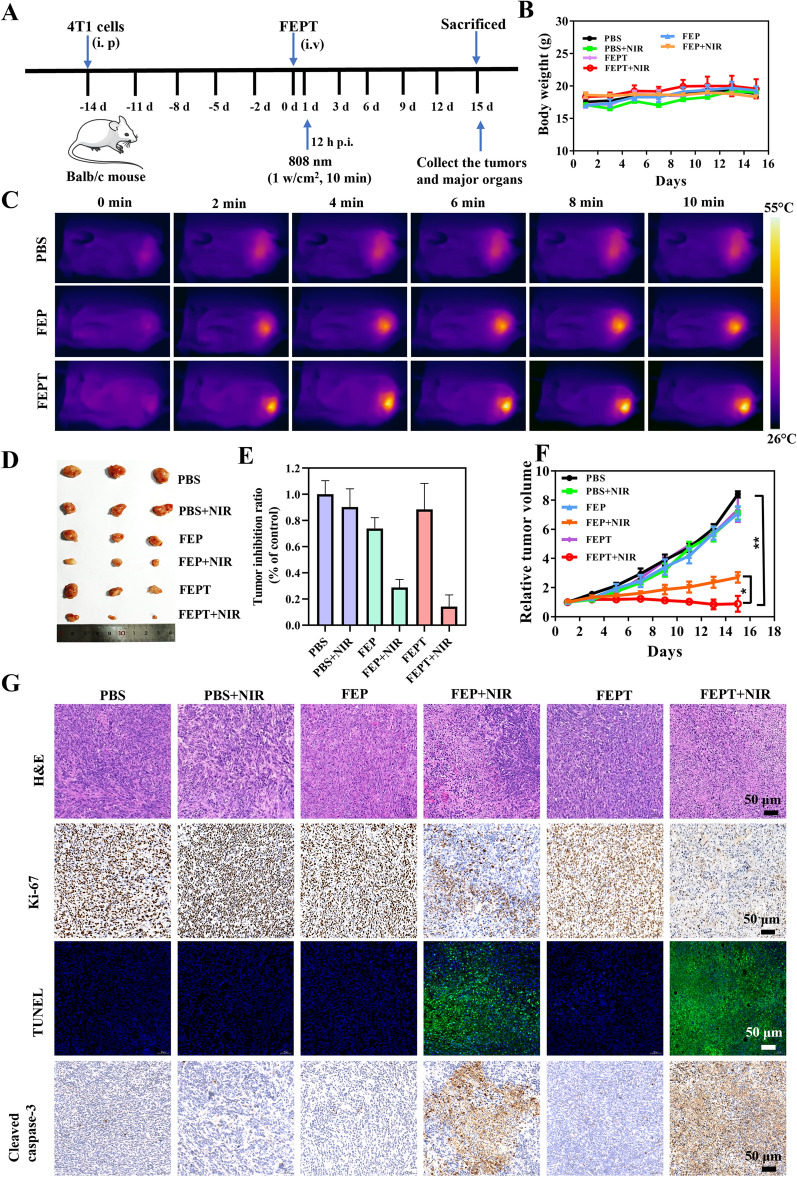
**A** The schematic diagram of 4T1 tumor-bearing mouse model and in vivo cancer therapy. **B** The body weights curves of mice in different groups. For irradiation groups, tumor regions were exposed to 808 nm laser at 1.0 W/cm^2^ for 10 min at 12 h post-injection. **C** IR thermal images of 4T1 tumor-bearing mice after intravenous injection of PBS, FEP or FEPT for 12 h and then treated with 808 nm laser irradiation (1.0 W/cm^2^, 10 min). **D** Photographs of tumors. **E** Tumor weights of different treated groups. **G** H&E, TUNEL, immunohistochemical analysis (IHC) of Ki-67 and cleaved caspase-3 in tumor tissues collected at 15 days post PTT/ PDT treatment. **F** Relative tumor growth curves of mice in different groups. V_0_: tumor volumes in the irradiated PBS-treated group at day 0, V: tumor volumes at different times. Positive signals in IHC staining are brown and the blue signal are nuclei. Apoptotic cells in TUNEL staining are green fluorescence, and the nuclei are stained with Hoechst33258. Scale bar: 50 μM. Error bars: mean ± SD (n = 3). *p < 0.05; **p < 0.01; ***p < 0.001

Most importantly, based on the aforementioned results, we further evaluated the effect of in vivo phototherapy with FEP or FEPT against cancers (Fig. 5A). Balb/c female mice (n = 3 per group, ~ 18 g each) were randomly divided into six groups. When the volumes of tumors grew to 100 mm^3^ approximately,_,_ FEP, FEPT (100 μl, 500 μM) and PBS (100 μl) were injected into the mice through the tail vein. After 12 h injection, the tumors in the irradiated groups (PBS + NIR, FEP + NIR and FEPT + NIR) were exposed to 808 nm laser irradiation (1.0 W/cm^2^) for 10 min. The temperature variation was monitored using photothermal imaging with an infrared thermal camera during the process of cancer therapy. As shown in Fig. 5C, the temperature increased rapidly by 22 °C approximately at the tumor sites in the FEPT-treatment group within 10 min under a single 808 nm irradiation. While, in the irradiated FEP group, the temperature increased was only  about14.4 °C, suggesting that FEPT has more excellent photothermal effect than the non-targeted FEP for cancer therapy. The tumor volumes of all the groups were measured every two days to monitor the tumor growth during the therapeutic process to estimate the outcome of cancer therapy. Without irradiation exposure, the tumor sizes of the FEP and FEPT-injected mice increased as quickly as that of PBS-injected mice. In contrast, the tumor growth in the irradiated FEP and FEPT groups was effectively inhibited. Notably, compared with FEP, FEPT was much better for inhibiting tumor growth (Fig. 5F). During the 15 days of treatments, there were no apparent changes in body weights in different groups (Fig. 5B), implying negligible toxicity of all treatments. After 15 days injection, mice were sacrificed, and the excised tumors as well as major organs were collected for imaged, H&E and IF staining. The excised tumor images (Fig. 5D) and weights (Fig. 5E) showed that the smallest sizes and the lightest weights of tumors were in the group of the irradiated FEPT, which proved the enhanced anticancer efficiency of FEPT under 808 nm laser irradiation. According to Additional file 1: Fig. S23, the H&E staining of major organ tissues in the irradiated FEPT-injected mice had no obvious difference in comparison to other groups after treatments, and these preliminary results demonstrated that FEPT could be used as an outstanding photosensitizer for efficient phototherapy in vivo. In addition, H&E, Ki-67, and TdT-mediated dUTP nick end labeling **(**TUNEL) assays were applied to assess morphological characteristics, cell proliferation and apoptosis in tumors, respectively (Fig. 5G). Compared with other groups, including the irradiated FEP, the morphology of tumor cells in the irradiated FEPT group was greatly changed that the cellular outline was ambiguous and the cell nuclei were absolutely disappeared. Besides, the tumor tissues of the irradiated FEPT group resulted in the lowest level of Ki-67 positive signals and the highest level of TUNEL positive signals in the apoptotic cells, indicating that the synergetic PTT and PDT can effectively induce cancer cell apoptosis by intravenous injection of mitochondria-targeted probe FEPT and irradiated with 808 nm laser.

### Cell apoptosis pathway of phototherapy in vivo

Since caspase-3 is an important biomarker in mitochondria-mediated intrinsic apoptosis [37], immunohistochemical analysis (IHC) of cleaved caspase-3 was conducted to verify that the mitochondria-targeting FEPT could enhance cell apoptosis with 808 nm laser irradiation. In comparison to other groups, the crucial fragmentation and disappearance of cell nucleias well as the remarkably increased activity of caspase-3 proved severe apoptosis in the tumorsof the mitochondria-targeting FEPT treated-mice after NIR irradiation (Fig. 5G). Combined with theaforementioned in vitro cellular experiments of cleaved caspase-3, the mechanism studies further indicated that the mitochondrial localization-based PDT and PTT induced by irradiated FEPT could result in mitochondrial dysfunction and apoptosis via caspase-3 pathway.

### Systemic cancer immunotherapy

**Fig. 6:**
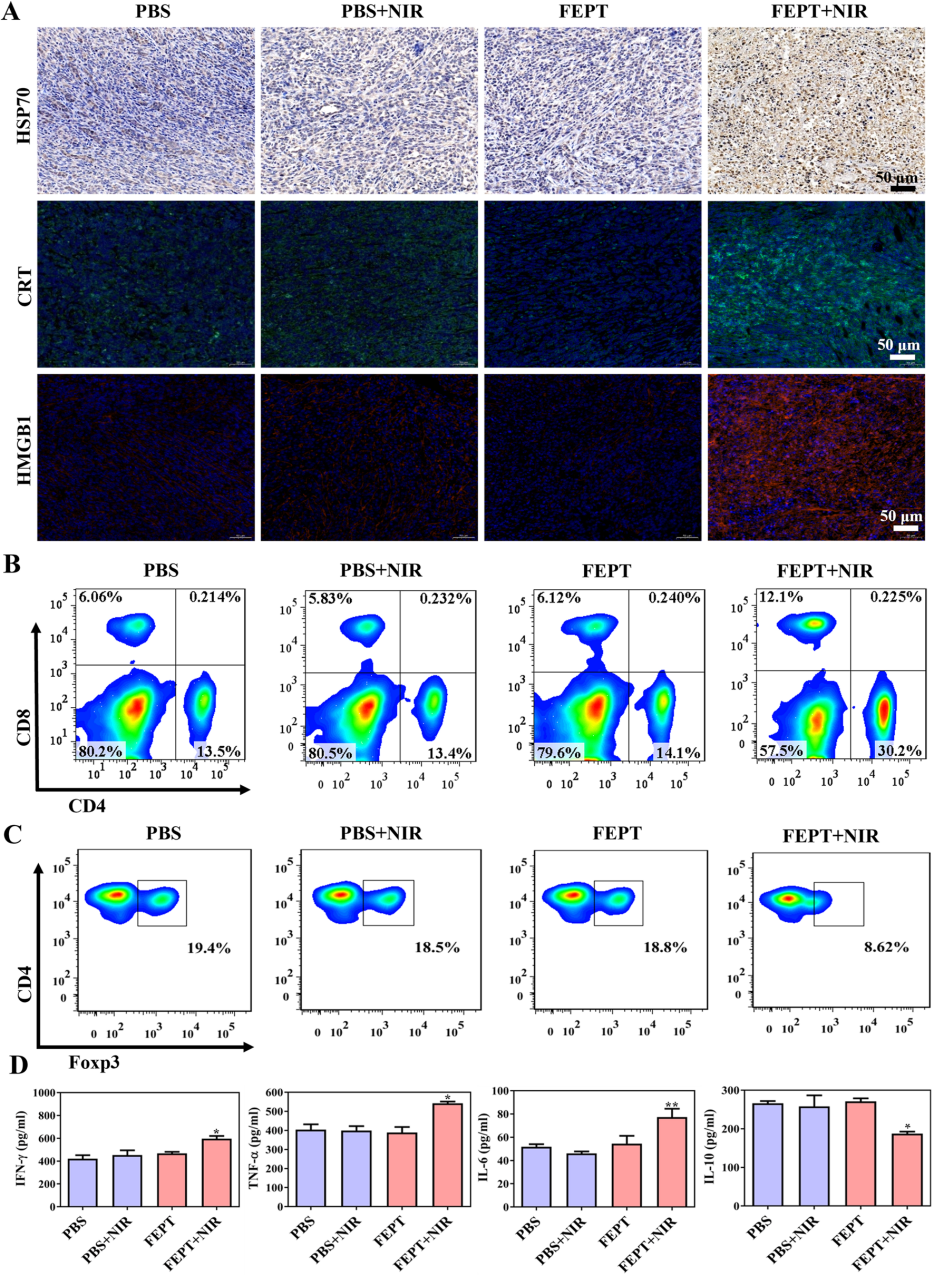
**A** IHC staining images of HSP70, IF staining images of CRT and HMGB1 in tumor tissues of mice. The blue signals in IHC and IF staining indicate cellular nuclei that stained with hematoxylin and Hoechst 33258, respectively. HSP70 positive signals in IHC are brown. Green or red fluorescence represents CRT or HMGB1, respectively. Scale bar: 50 μM. **B-C** Representative results of CD4^+^T and CD8^+^T cells as well as Tregs in splenic lymphocytes isolated from the various treated mice. **D** Typical cytokines in serum from different-treated mice. Error bars: mean ± SD (n = 3) *p < 0.05; **p < 0.01; ***p < 0.001

Based on the mentioned above in vitro and in vivo experiments, FEPT was superior to FEP in cancer therapy. To verify the high level of ROS and hyperthermia induced by irradiated FEPT whether triggers the ICD that induced by dying cancer cells, biomarkers of damage-associated molecular pattens (DAMPs) were analyzed. The typical biomarkers of DAMPs include the cell surface exposure of heat-shock proteins (HSP70, HSP90) and calreticulin (CRT), the extracellular release of high-mobility group box-1 (HMGB1), type I interferons (IFNs), adenosine triphosphate (ATP), and members of the IL-1 cytokine family [38, 39]. Herein, CRT, HSP70, and HMGB1 were selected to evaluate the anticancer effects. As the “eat me” signal, CRT can migrate from the endoplasmic reticulum to the cell membrane to promote the cell uptake by antigen-presenting cells (APCs), thereby activating the immune response [40]. HSP70, released by necrotic cells, has significant immunogenic potential for eliciting strong T cell response upon being bound to cancer antigen or antigen-free [41]. As shown in Fig. 6A, IHC staining of HSP70 and IF staining of CRT manifested that the PBS-treated and the unirradiated FEPT groups exhibited mild expression of HSP70 and CRT on the cell surface without NIR irradiation. By contrast, the 4T1 cells treated with irradiated FEPT upregulated higher expression levels of HSP70 and CRT. Moreover, the extracellular release of HMGB1 is also a vital biomarker of DAMPs. Upon migrating from the nucleus to the extracellular environment, HMGB1 can serve as a “danger” signal molecule to activate the immune response. The release of HMGB1 was significantly increased in the TME of the tumors in the irradiated FEPT group. The PBS-treated and the unirradiated FEPT groups with weak fluorescent signals in the TME suggested that HMGB1 did not migrate in those groups. Inspiringly, with 808 nm laser irradiation, the fluorescence intensity of HMGB1 in the FEPT group was brighter than that of other groups. All those results demonstrated that the NIR-II molecular phototheranostic agent FEPT with PDT and PTT can efficiently boost ICD to amplify cancer therapy efficacy.

Based on the high-level release and relocation of DAMP biomarkers, the effector T cells, such as helper T lymphocytes (CD4^+^T cells, Ths), cytotoxic T lymphocytes (CD8^+^ T cells, CTLs), regulatory T cells (Tregs) in spleens and tumors as well as the typical cytokines in serum of different groups, were measured to confirm that whether the mitochondria-targeting phototherapeutic FEPT could promote systemic immune response. The representative flow cytometry results showed that the proportions of both CD4^+^T and CD8^+^T cells in irradiated FEPT-treated mice were remarkably higher than that in other groups. For example, both CD4^+^T and CD8^+^T cell proportions were nearly twice higher than those in only PBS-treated mice (Fig. 6B). Compared with the unirradiated PBS group, the proportion of Tregs in the irradiated FEPT group decreased by about 55% (Fig. 6C). Quantitative results of CD3^+^CD4^+^T cells, CD3^+^CD8^+^T cells, and Tregs in CD4^+^T cells in splenic lymphocytes isolated from the various treated mice was shown in Additional file 1: Fig. S24. Analogously, the IF staining of CD4^+^T, CD8^+^T and IHC of Tregs were further conducted in the spleen and tumor tissues (Additional file 1: Fig. S25). The positive signals of CD4^+^T and CD8^+^T cells in the irradiated FEPT-treated mice were also remarkably higher than that in other groups, and induced the lowest Treg rates in spleen and tumor tissues. Additionally, a large amount of anticancer-involved cytokines including interferon-γ (IFN-γ), interleukin-6 (IL-6), and tumor necrosis factor-α (TNF-α) were obviously increased. On the contrary, the pro-cancer immune cytokines, such as interleukin-10 (IL-10), was decreased significantly (Fig. 6D), which was consistent with the aforementioned results. Therefore, the highly efficient activation of the immune system was achieved by the outstanding fluorophore FEPT. On account of the *in-situ* enhancement efficacy in PTT and PDT via simultaneous mitochondria-targeting, the phototheranostic agent FEPT was efficient in amplifying ICD in vivo to enhance the therapeutic effect.

## Conclusions

In summary, we newly developed a mitochondria-targeting molecular phototheranostic platform that can simultaneously amplify PDT and PTT, and trigger effective ICD by boosting T cell-mediated anticancer immune response. The rationally designed mitochondria-targeting FEPT showed the excellent generation of ROS and hyperthermia. In vivo experiments further prove that the FEPT probe has the merits of outstanding in vivo imaging ability, good biocompatibility, and excellent antitumor ability. Especially, owing to the *in-situ* amplification in both PDT and PTT by mitochondria-targeting, FEPT can effectively boost immune response, and significantly increase the proportions of CD4^+^ T cells, CD8^+^ T cells as well as the decreased Tregs proportion, demonstrating an remarkable immunotherapy efficacy on cancer ablation. Therefore, we believed that FEPT provides a mitochondria-targeting phototheranostic platform for imaging-guided synergistic PTT/PDT/IMT for cancer therapy.

## Experimental section

### Materials and reagents

Phosphate buffer (PBS), 9,10-Anthracenediyl-bis(methylene) dimalonic Acid (ABDA), 2,7-Dichlorodihydrofluorescein diacetate (DCFH-DA), Hoechst 33258 and all the ELISA Kits were purchased from Sigma-Aldrich Co., Ltd. Mito-Tracker Green FM (MTG) was bought from Beyotime Biotechnology Co., Ltd. AM/PI double stain kit and Cell Counting Kit (CCK8) were bought from Dojindo Co.,Ltd. Mitochondria Staining Kit (JC-1) was purchased from Multiscience (Lianke) Biotech, Co., Ltd. All the used antibodies were purchased from Biolegend Co., Ltd.. The 4T1 cell (mouse mammary carcinoma cell) and HC11 ( mouse mammary epithelial cell) were bought from American Type Culture Collection (ATCC) and cultured in Dulbecco’s modified Eagle’s medium (DMEM, Hyclone) with 10% fetal bovine serum (FBS, ExCell Bio, Shanghai, China) at 37 °C and in a 5% CO_2_ humidified air environment. The confocal dish was purchased from Cellvis, Mountain View, CA. All the chemicals were used without further purification.

### Characterizations

Ultra violet-visible-near infrared (UV-Vis-NIR) absorption spectra were measured by a MAPADA UA-3200S spectrometer (China). Hydrodynamic diameters and zeta potentials were measured using a Malvern Nano-ZS Particle Sizer (Malvern Instruments, Southborough, UK). Transmission electron microscopy (TEM) was carried out on a JEM-100CXII electron microscope. Fluorescence spectra measurements were performed with FLS-980 Edinburgh Fluorescence Spectrometer. Temperature evolution curves were tested by an infrared thermal imaging camera (Fotric 225 s, China) upon irradiation with an 808 nm laser (1.0 W/m^2^, MDL-XF-808 nm, 10.0 W, Changchun New Industries Optoelectronics Technology Co., Ltd.).

### Photothermal heating

A NIR laser (808 nm, 1.0 W/cm^2^) was used to stimulate the different concentration of FEPT (10, 25, 50,75, and 100 μM) in an aqueous solution. The photothermal curves and images that during laser irradiation were recorded every two minutes using an infrared camera. The photothermal effects of FEPT (50 μM) under 808 nm laser irradiation with different power densities (0, 0.25, 0.5, 0.75, 1, 1.25, and 1.5 W/cm^2^) were evaluated in the same way. Then temperature elevation of FEPT (50 µM) in an aqueous solution under five irradiation-cooling cycles (808 nm, 1.0 W/cm^2^) was carried out. In addition, the temperature elevation of FEP (50 µM) in an aqueous solution under one irradiation-cooling cycle (808 nm, 1.0 W/cm^2^) was also carried out for calculating its PCE.

### Detection of singlet oxygen production

ABDA was used to detect the singlet oxygen production. The ABDA probe solution (40 µM) was mixed with FEP or FEPT solution (50 µM), followed by 808 nm laser irradiation (1.0 W/ cm^2^, 0, 2.5, 5, 7.5, and 10 min). The UV–Vis absorption spectra were recorded at different time points and the decrease rate of absorption at 380 nm is proportional to the singlet oxygen production.

### Cytotoxicity and in vitro cancer efficiency

The in vitro anticancer effect was measured by CCK8 assay. 4T1 cells were seeded in 96-well plates at a density of 5 × 10^3^ cells per well and incubated for 24 h. Afterward, cells were incubated with different concentrations of FEP or FEPT for 4 h. Then, the 4T1 cells were exposed to 808 nm laser radiation (1.0 W/cm^2^) for 10 min. At the end of the incubation, 10 μl CCK8 solution was added, and the plate was incubated for another 2 h. Finally, the absorbance values of the cells were determined by using a microplate reader at 450 nm. The background absorbance of the well plate was measured and subtracted. The cytotoxicity was calculated by dividing the optical density (OD) values of the treated groups (T) by the OD values of the control (C) (T/C × 100%). The group without FEPT and laser irradiation was used as the control group.

### Cancer therapy in vivo

To investigate the anticancer effect of the FEPT, Balb/c female mice were subcutaneously injected with 2 × 10^6^ 4T1 cells. When the tumor volumes reached 100 mm^3^, tumor-bearing mice were divided randomly into six groups (n = 3), and FEP and FEPT (100 µl, 500 µM) were intravenously injected into mice through the tail veil. After 12 h injection, tumor sites were exposed to 808 nm laser irradiation (1.0 W/ cm^2^) for 10 min. Thermal imaging was conducted by an infrared thermal imaging camera. Mice body weights and tumor volumes were monitored every two days. The tumor volume was calculated by the following equation: Volume = length × width^2^/2. After 15 days of treatment, all the mice were sacrificed for histological analysis.

### Statistical analysis

All the data were expressed as the mean ± SD. Differences between two groups were analyzed by a two-tailed student’s test. These analyses were carried out using SPSS 26.0. *p < 0.05; **p < 0.01, ***p < 0.001.

## Supplementary Information

**Additional file 1: Fig. S1.** Synthetic routes of FEPT and FEP. **Fig. S2.** 1H NMR of compound 2. **Fig. S3.** ^13^C NMR of compound 2. **Fig. S4.** ^1^H NMR of compound 5. **Fig. S5.** ^13^C NMR of compound 5. **Fig. S6**^. 1^H NMR of compound IR-FE. **Fig. S7.** ^13^C NMR of compound IR-FE. **Fig. S8.** ^1^H NMR of compound 8. **Fig. S9.** ^13^C NMR of compound 8. **Fig. S10.** ^1^H NMR of compound FEPT. **Fig. S11.** ^13^C NMR of compound FEPT. **Fig. S12.** ^13^P NMR of compound FEPT. **Fig. S13.** HRMS of compound FEPT. **Fig. S14.** ^1^H NMR of compound FEP. **Fig. S15.** ^13^C NMR of compound FEP. **Fig. S16.** HRMS of compound FEP. **Fig. S17. A** UV–Vis absorbance spectra of FEP and FEPT. **B** UV–Vis absorbance of FEP (black line) and NIR-II fluorescence emission of FEP (red line) with a peak at 1085 nm under 808 nm laser excitation (solvents: water). **C** The fluorescence emission of FEP (red line) and FEPT (black line) under 405 nm laser excitation (solvents: water). **D** Photothermal conversion performance curves of FEP. **Fig. S18. A** UV–Vis absorbance stability of FEPT in different PH resolution. **B** NIR-II fluorescence stability of FEPT with a peak at 1085 nm under 808 nm laser excitation (solvents: different PH solutions). **C** The size of FEPT measured by dynamic light scattering (solvents: water). **D** The TEM of FEPT in water. Scale bar:100 nm. **Fig. S19.** Cellular uptake images of FEPT in 4T1 cells captured by LSM980. Scale bar: 20 μm. **Fig. S20. A** Mean fluorescence intensity of ROS in 4T1 cells detected with DCFH-DA probe after different treatments. **B** Changes in mitochondrial membrane potential (ΔΨm) of the relative fluorescent intensity of JC-1 aggregates and monomer in 4T1 cells with different treatments. **C** Quantitative fluorescence intensity of cleaved caspase-3 in 4T1 cells with different treatments analyzed by CLSM. **D** Cleaved caspase-3 detection of 4T1 cells after different treatments by ELISA. Error bars: mean ± SD (n = 3). *P < 0.05; **P < 0.01; ***P < 0.001. **Fig. S21**. H&E staining images of five major organs (heart, liver, spleen, lung and kidney) in the healthy Balb/c mice after intravenous injection of PBS, FEP and FEPT without 808 nm laser irradiation at 15 d  post-injection. Scale bar: 50 μm. **Fig. S22. A** Blood routine examinations in the PBS, FEP and FEPT groups. **B** Chemistry indexes of the liver and renal function tests in the PBS, FEP and FEPT groups. All the tests are detected in the healthy Balb/c mice without NIR irradiation at 15 d post-injection. **Fig. S23**. H&E staining images of five major organs (heart, liver, spleen, lung, and kidneys) in 4T1 tumor-bearing mice with different treatments at 15 d post-injection. Scale bar: 50 μm. **Fig. S24**. Quantitative results of CD3^+^CD4^+^T cells, CD3^+^CD8^+^T cells, and Tregs in CD4^+^T cells in splenic lymphocytes isolated from different-treated mice. Mean ± SD (n = 3). *P < 0.05; **P < 0.01; ***P < 0.001. **Fig. S25**. The IHC analysis of Tregs and the IF analysis of infiltrating CD4^+^T (green) and CD8^+^T (red) cells in the spleen and tumor tissues. The blue signals in IHC and IF staining indicate cellular nuclei that stained with hematoxylin and Hoechst 33258, respectively. The positive signal of Tregs in IHC staining is brown. Green and red fluorescence signal indicates CD4^+^T, CD8^+^T cells, respectively. Scale bar: 50 μm.

## Data Availability

All data generated or analyzed during this study are included in this article.

## Abbreviations

NIR: Near-infrared
PTT: Photothermal therapy
PDT: Photodynamic therapy
IMT: Immune therapy
TME: Tumor microenvironment
DC: Dendritic cells
PCE: Photothermal conversion efficiency
BBTD: Benzobisthiadiazole
EDOT: 3,4-Ethylenedioxy thiophene
ROS: Reactive oxygen species
ICD: Immunogenic cell death
PSs: Photosensitizers
MMP: Mitochondrial membrane potential
TPP: Triphenyl phosphonium
DQA: Dequalinium
CLSM: Confocal laser microscope system
QY: Quantum yield
NMR: Nuclear magnetic resonance
HRMS: High-Resolution Mass Spectrometry
ABDA: 9,10-Anthracenediyl-bis (methylene) dimalonic Acid
MTG: Mito-tracker Green
PCCs: Pearson’s correlation coefficients
DCFH-DA: 2,7-Dichlorodi-hydrofluorescein diacetate
DCFH: Dichlorodihydrofluorescein
DCH: 2′,7′-Dichlor
IF: Immunofluorescence
H&E: Hematoxylin-eosin
ALP: Alkaline phosphatase
AST: Aspartate aminotransferase
ALT: Alanine transaminase
γ-GT: γ-Glutamyltranspeptidase
CREA: Creatinine urea
UA: Uric acid
TUNEL: TdT-mediated dUTP nick end labeling assays
IHC: Immunohistochemical analysis
Ths: Helper T lymphocytes
CTLs: Cytotoxic T lymphocytes
Tregs: Regulatory T cells
DAMPs: Damage-associated molecular pattens
HSP70: Heat-shock proteins 70
CRT: Calreticulin
HMGB1: High-mobility group box-1
ATP: Adenosine triphosphate
IFN-γ: Interferon-γ
IL-6: Interleukin-6
TNF-α: Tumor necrosis factor-α
IL-10: Interleukin-10
