# Supplementary material for: A mitochondria-targeted molecular phototheranostic platform for NIR-II imaging-guided synergistic photothermal/photodynamic/immune therapy
Source: J Nanobiotechnology. 2022 Nov 11;20:475. doi: 10.1186/s12951-022-01679-0 (PMC9652787; doi:10.1186/s12951-022-01679-0)
Supplement: Supplementary file 1 — Additional file 1: Fig. S1. Synthetic routes of FEPT and FEP. Fig. S2. 1H NMR of compound 2. Fig. S3. 13C NMR of compound 2. Fig. S4. 1H NMR of compound 5. Fig. S5. 13C NMR of compound 5. Fig. S6. 1H NMR of compound IR-FE. Fig. S7. 13C NMR of compound IR-FE. Fig. S8. 1H NMR of compound 8. Fig. S9. 13C NMR of compound 8. Fig. S10. 1H NMR of compound FEPT. Fig. S11. 13C NMR of compound FEPT. Fig. S12. 13P NMR of compound FEPT. Fig. S13. HRMS of compound FEPT. Fig. S14. 1H NMR of compound FEP. Fig. S15. 13C NMR of compound FEP. Fig. S16. HRMS of compound FEP. Fig. S17. A UV–Vis absorbance spectra of FEP and FEPT. B UV–Vis absorbance of FEP (black line) and NIR-II fluorescence emission of FEP (red line) with a peak at 1085 nm under 808 nm laser excitation (solvents: water). C The fluorescence emission of FEP (red line) and FEPT (black line) under 405 nm laser excitation (solvents: water). D Photothermal conversion performance curves of FEP. Fig. S18. A UV–Vis absorbance stability of FEPT in different PH resolution. B NIR-II fluorescence stability of FEPT with a peak at 1085 nm under 808 nm laser excitation (solvents: different PH solutions). C The size of FEPT measured by dynamic light scattering (solvents: water). D The TEM of FEPT in water. Scale bar:100 nm. Fig. S19. Cellular uptake images of FEPT in 4T1 cells captured by LSM980. Scale bar: 20 μm. Fig. S20. A Mean fluorescence intensity of ROS in 4T1 cells detected with DCFH-DA probe after different treatments. B Changes in mitochondrial membrane potential (ΔΨm) of the relative fluorescent intensity of JC-1 aggregates and monomer in 4T1 cells with different treatments. C Quantitative fluorescence intensity of cleaved caspase-3 in 4T1 cells with different treatments analyzed by CLSM. D Cleaved caspase-3 detection of 4T1 cells after different treatments by ELISA. Error bars: mean ± SD (n = 3). *P < 0.05; **P < 0.01; ***P < 0.001. Fig. S21. H&E staining images of five major organs (heart, liver, spleen, lung and kidney) [file 12951_2022_1679_MOESM1_ESM.pdf]

Additional file 1

A mitochondria-targeted molecular phototheranostic platform for NIR-II imaging-guided synergistic photothermal /photodynamic /immune therapy

Sha Yang[1,2], Gui-long Wu[1], Na Li[1], Minghui Wang[1], Peixian Wu[1], Yuxuan He[1], Wei Zhou[1], Hao Xiao[1], Li Tang[1,3]*, Xiaofeng Tan[1]*, Qinglai Yang[1]*

1.Center for Molecular Imaging Probe, Hunan Province Key Laboratory of Cancer Cellular and Molecular Pathology, Cancer Research Institute, Hengyang Medical School, University of South China, Hengyang, Hunan 421001, China.

2. Cancer Pathology Research group & Department of Pathology, Xiangnan University, Chenzhou, Hunan 423099, China.

3. Key Laboratory of Tropical Medicinal Plant Chemistry of Ministry of Education, College of Chemistry and Chemical Engineering, Hainan Normal University, Haikou, Hainan 571158, China.

***Correspondence:** tanxiaofeng@usc.edu.cn (X. Tan), tanglicq@outlook.com (L. Tang), qingyu513@usc.edu.cn (Q. Yang).

Instruments

^{1}H and ^{13}C NMR spectra were performed on 500 MHz NMR spectrometers (Bruker AVANCE) using $CDCl_3$. Mass spectra were in general recorded on QSTAR Elite (ABI). High-resolution mass spectra (HRMS) were performed on a Bruker micrOTOF-Q II mass spectrometer. Fluorescence imaging (FI) was performed at a Xenogen IVIS Lumina system (Caliper Life Sciences). Confocal laser scanning microscopy images were collected with a confocal laser scanning microscope (Zeiss LSM980, Germany). Flow cytometry analysis was performed using a CytoFLEX flow cytometer (Beckman Coulter, Fullerton, CA, USA).

Quantum yield test

The fluorescence quantum yield of the fluorophores was measured in a similar way to the previous method.[1] The fluorescence spectra in the region of 900-1800 nm were measured by an array detector (Princeton OMA-V) and a spectrometer (Acton SP2300i) under an 808 nm diode laser excitation (RMPC lasers, 160 mW). The fluorescence quantum yield was determined against the reference fluorophore IR-FEP with a known quantum yield of 2.0% (Φ_{st}) in an aqueous solution.[2] All samples were measured at 25°C with an optical density (OD) of 0.1 at 808 nm. Their NIR-II fluorescence emission intensities were measured under the 808 nm excitation. Using the measured OD at 808 nm and spectrally integrated fluorescence intensity. η represents the refractive index of the solvent, and the quantum yield of the test sample can be calculated according to the following equation:

$$QY_{\mathrm{sample}} = QY_{\mathrm{ref}} \times \frac{slope_{\mathrm{sample}}}{slope_{\mathrm{ref}}} \times \frac{\eta_{\mathrm{sample}}^2}{\eta_{ref}^2}$$

Both η_{sample} and η_{ref} are the refractive index of water。

Photothermal conversion efficiency

The photothermal conversion efficiency was calculated using the following equation: where h is the heat transfer coefficient, s is the surface of the container, T_{max} and T_{surr} are the equilibrium temperature and ambient temperature, respectively. Q_0 is the heat associated with the light absorbance of the solvent, A_λ is the absorbance of FEPT at 808 nm, and I is the laser power density. According to the above equation, the η value of FEPT was determined to be about 56.8%. The η was calculated by equation:[3]

$$\eta = \frac{\mathrm{hs}(T_{\mathrm{max}} - T_{\mathrm{surr}}) - Q_0}{I(1-10^{-A_\lambda})} \qquad (1)$$

hs can be calculated by the follow equation:

$$\mathrm{hs} = \frac{\sum m_i C_{P.i}}{\tau_s} \quad (2)$$

$$\tau_s = \frac{t}{-\ln\theta} \quad (3)$$

$$\theta = \frac{T - T_{surr}}{T_{max} - T_{surr}} \quad (4)$$

$$Q_0 = hs(T_{max} - T_{surr}) \quad (5)$$

h represents the heat transfer coefficient,

s represents the sample container surface area,

T_{max} represents the steady-state maximum temperature,

T_{surr} represents the ambient room temperature,

T represents instantaneous temperature during cooling,

t represents the time it takes by T cooled to room temperature,

C approximate to the specific heat capacity of water,

m represented the mass of the solution (g),

Q_0 represents the energy input by the same solvent without NPs in the same quartz cuvette after the same laser irradiation.

In vitro cellular uptake

For cell uptake study, the 4T1 cells (1 × 10^5 cells per dish) were cultured in a confocal dish with DMEM medium containing 10% fetal bovine serum (FBS, Gibco, Invitrogen, Carlsbad, CA, USA), 100 IU/ml penicillin, 100 μg/ml streptomycin for 24 h and then substituted with the fresh medium contained FEPT (50 μM). Followed by the incubation for different times (0, 2, 4, and 6 h), and the suspension cells were collected. Then the cells were washed twice with PBS and the fluorescence of 4T1 cells were collected with a confocal laser scanning microscope (Zeiss LSM980, Germany). The excitation wavelength of FEPT was 405 nm.

Co-staining imaging in living cells

After the 4T1 cells (5 ×10^4 cells per dish) were plated on chamber slides for 12 h, 50 μM of FEP or FEPT was added into the medium. After 4 h of incubation, the cells were incubated with Mito-Tracker Green FM (MTG) (200 nM) for 30 min. Finally, the fluorescence imaging was performed. The excitation wavelength of MTG and FEP or FEPT were 488 nm and 405 nm, respectively.

Monitoring of intracellular ROS generation

ROS generation inside cells under laser irradiation was detected with DCFH-DA as an indicator. 4T1 cells were cultured in a 35 mm confocal dish (glass bottom dish) at an appropriate density for 12 h. Following the incubation with FEP or FEPT (50 μM) for 4 h in the dark, DCFH-DA (1 × 10^{-5} M) was loaded into the cells. After 30 min of incubation, cells were washed twice with PBS and then irradiated under 808 nm laser (1.0 W/cm^2) for 10 min. After another 30 min of incubation at 37 °C, the fluorescence images of treated

cells were acquired by LSM 980. The DCFH-DA was excited at 488 nm. No background fluorescence of cells was detected under the setting condition.

Monitoring changes of mitochondria transmembrane potential

A Mitochondria staining kit (JC-1) was employed to investigate the mitochondria transmembrane potential of the cells with different treatments. 4T1 cells (1×10^5 cells per dish) were cultured in confocal dishes and divided into six groups: PBS, PBS+NIR, FEP, FEP+NIR, FEPT and FEPT+NIR. Cells were first incubated with different substrates for 4 h, respectively, and the concentration of FEP or FEPT was 50 μM. The irradiation groups were irradiated with laser at 1.0 W/cm^2 for 10 min. After further incubation for 4 h, JC-1 (2 μM) solution was added and incubated for 10 min under dark, and the confocal images were recorded with LSM980 after washed with PBS. The excitation wavelength of JC-1 was 488 nm.

Calcein-AM/PI Staining of live/dead cell induced by laser irradiation

4T1 cells (1×10^5 cells per dish) were seeded and cultured in a confocal dish with 2.0 ml of medium. 4T1 cells were pre-incubated with FEP or FEPT (50 μM) for 4 h and then exposed to 808 nm laser irradiation for 10 min. After incubated for another 1 h, the cells were stained with a mixed solution of PI (8 μM) and Calcein-AM (2 μM) for 30 min in the PBS. After being washed gently with PBS, the cells were then imaged by LSM980. The excitation wavelength was 488 nm for Calcein-AM, and 543 nm for PI.

Evaluation of cleaved caspase-3 with IF and ELISA

The immunofluorescence (IF) staining was performed to evaluate the cleaved caspase-3

expression of 4T1 cells with different treatments. The cells were treated with blank medium and medium containing FEP or FEPT (50 μM) for 4 h. Then the cells in irradiated groups were treated with 808 nm laser irradiation (1.0 W/cm^2, 10 min) and then fixed with 4% PFA for 20 min at 4 °C and treated with 0.2% Triton X-100-PBS for exact 5 min. BSA-PBS was used to block the cells for 1 h at room temperature. Subsequently, the cells were stained with anti-cleaved caspase-3 primary antibody, secondary antibody and Hoechst 33258. Finally, the cells were evaluated by confocal fluorescence imaging (Ex = 488 nm). ELISA was further performed with Mouse cleaved caspase-3 ELISA Kit. 4T1 cells (2×10^5 cells per well) were seeded in six-well plates overnight. Then, a fresh medium containing FEP or FEPT was used to replace the medium. After 4 h of incubation, the cells were irradiated using an 808 nm laser (1.0 W/cm^2, 10 min). Afterward, the medium was discarded and washed with cold PBS. Protein lysis buffer (100 μl, containing 1 mM PMSF) was added to each well, and the cells were grinded for 30 min. The whole process was operated on ice. After 30 min, all samples were centrifuged to collect the supernatant (13000 rpm, 15 min, 4 °C). Thereafter, the collected proteins were measured with Mouse cleaved caspase-3 ELISA Kit.

Animal tumor models

Balb/c mice (female, 4-5 weeks old) were obtained from Hunan SJA Laboratory Animal Co., Ltd. 100 μl 4T1 cell suspension (2×10^6 cells) were subcutaneous injected into each mouse to establish the cancer models. All animal experiments were approved by University of South China Animal Experiment Ethics Review and the Health Guide for the Care and Use of Laboratory Animals of National Institutes. All mice received care in accordance with international ethics guidelines.

Systemic toxicity assessment

To evaluate the systemic toxicity, the mice were divided into three groups, including the groups of PBS (100 μl), FEP (100 μl, 500 μM) and FEPT (100 μl, 500 μM). *In vivo* blood biochemistry and blood routine analysis were comprehensively carried out to study the long-term biotoxicity of FEP or FEPT. Blood from three groups was collected on day 15. The hepatic and renal function parameters, including alkaline phosphatase (ALP), aspartate aminotransferase (AST), alanine transaminase (ALT), γ-glutamyltranspeptidase (γ-GT), creatinine (CREA), urea (UREA) and uric acid (UA) were monitored. For the blood routine examination, relative indexes such as white blood cells (WBC), red blood cells (RBC), hematocrit (HCT), hemoglobin (HGB), platelets (PLT), mean corpuscular volume (MCV), mean corpuscular hemoglobin (MCH), and mean corpuscular hemoglobin concentration (MCHC) were evaluated. Blood routine examinations and biochemical indexes were examined by hematology analyzer.

Histological analysis

After the treatment, major organs (heart, liver, spleen, lung, and kidney) and tumors of all mice were harvested. The $CD4^+$ T cells (stained with APC Anti-Mouse CD3 Antibody and FITC Anti-mouse CD4 Antibody), $CD8^+$ T cells (stained with APC Anti-Mouse CD3 Antibody and PE Anti-Mouse CD8a Antibody) and regulatory T cells (stained with FITC Anti-Mouse CD4 Antibody and APC Anti-Mouse Foxp3 Antibody) in spleens were detected by flow cytometry. Cytokines in serum were evaluated with ELISA. Tissues were washed with PBS, and fixed with paraformaldehyde for histology analysis. For IHC staining, after the tumor tissues were weighed, they were fixed in 4% neutral buffered formalin, processed routinely into paraffin, and sectioned at 4 μm. The tumor tissues were

stained with a primary antibody with Rabbit anti-mouse Ki-67, cleaved caspase-3, HSP70, and Foxp3 antibodies at 4 °C overnight, respectively. Besides, spleen slices were also fixed and stained with Rabbit anti-mouse Foxp3 antibodies. After washing three times with cold PBS buffer, HRP-conjugated secondary antibodies (1:1000) were added and incubated for 2 h at room temperature. Finally, added TMB resolutions for color reactions. The IF staining of CRT, HMGB1, $CD4^{+}T$ and $CD8^{+}T$ in tumor or spleen tissues as well as *Terminal dUTP Nick End Labeling* (TUNEL) staining in tumor or spleen tissues were also performed according to the standard protocols after being fixed. In the IHC and IF staining, the cellular nuclei were stained with hematoxylin and Hoechst 33258, respectively. The sections were eventually scanned with *Pannoramic DESK, P-MIDI, P250, P1000* (3D HISTECH, HUN).

Synthetic procedures and characterization of NIR-II fluorophores

Synthesis of FEPT and FEP

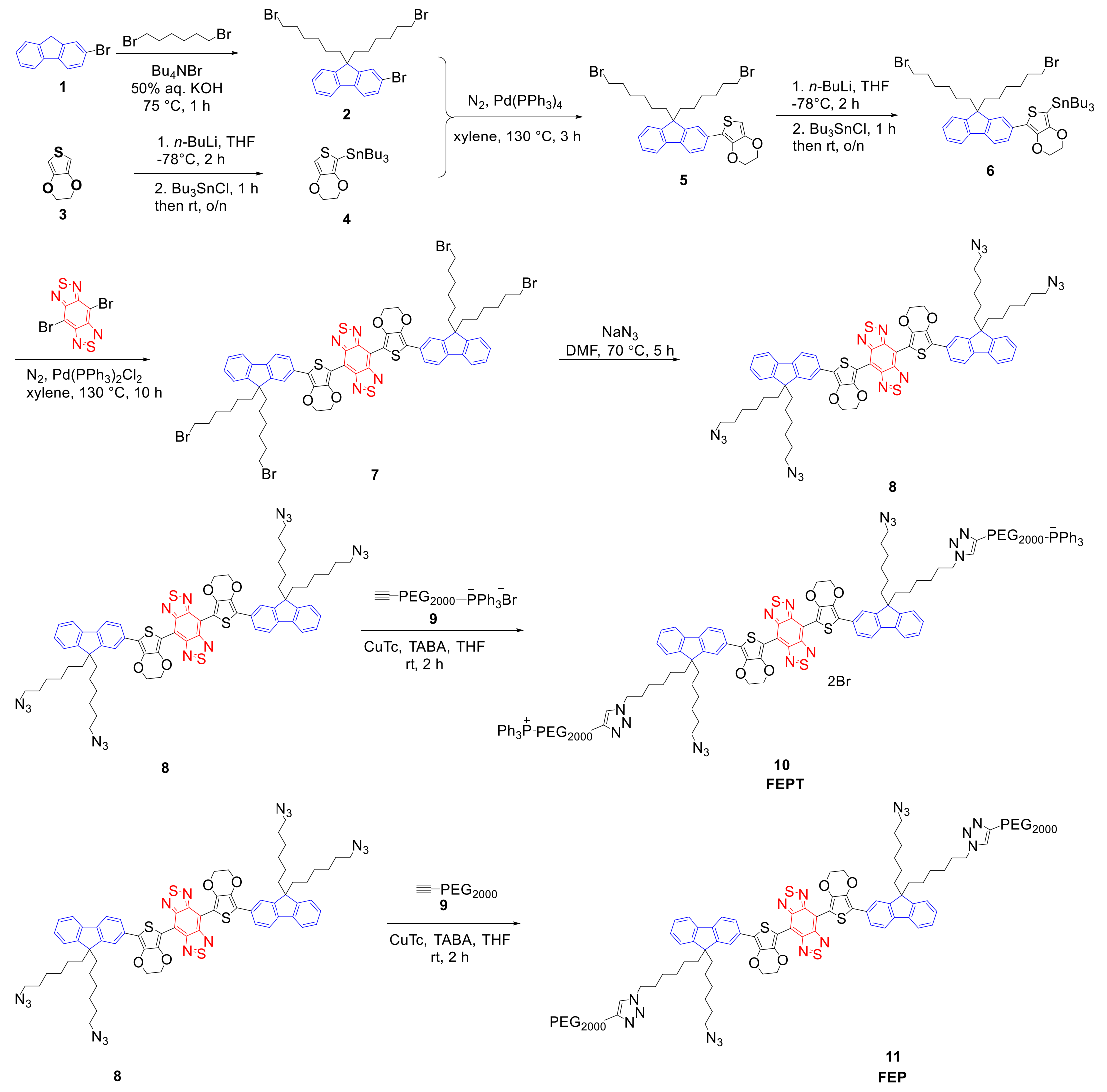

Fig. S1 Synthetic routes of FEPT and FEP.

2-bromo-9,9-bis(6-bromohexyl)-9H-fluorene (2): To 150 ml of 45% aqueous potassium hydroxide was added 6.0 g (24.4 mmol) 2-bromofluorene, 59.3 g (0.244 mol) 1,6-dibromohexane and 0.78 g (2.4 mmol) tetrabutylammonium bromide at 75°C. The mixture was stirring for 1 h, and then cooled down to room temperature. The aqueous layer was

extracted with dichloromethane. The organic layer was washed with 1.0 M aqueous HCl, then brine and water, and dried over anhydrous magnesium sulfate. After removal of the solvent and the excess 1,6-dibromohexane under reduced pressure, the residue was purified by column chromatography on silica gel (eluent petroleum ether) to afford yellow, which was further purified by recrystallization by petroleum ether under -20 °C to give white solid product 8.79 g (63%). ^{1}H NMR (500 MHz, $CDCl_3$). δ (ppm): δ 7.67 – 7.64 (m, 1H), 7.57 – 7.53 (m, 1H), 7.47 – 7.42 (m, 2H), 7.34 – 7.30 (m, 3H), 3.27 (t, *J* = 6.8 Hz, 4H), 2.00 – 1.89 (m, 4H), 1.69 – 1.61 (m, 4H), 1.23 – 1.15 (m, 4H), 1.10 – 1.02 (m, 4H), 0.66 – 0.54 (m, 4H). ^{13}C NMR (125 MHz, $CDCl_3$). δ (ppm): 152.59, 149.93, 140.16, 140.03, 130.05, 127.59, 127.10, 126.06, 122.80, 121.11, 121.05, 119.84, 55.24, 40.11, 33.86, 32.60, 28.98, 27.74, 23.46.

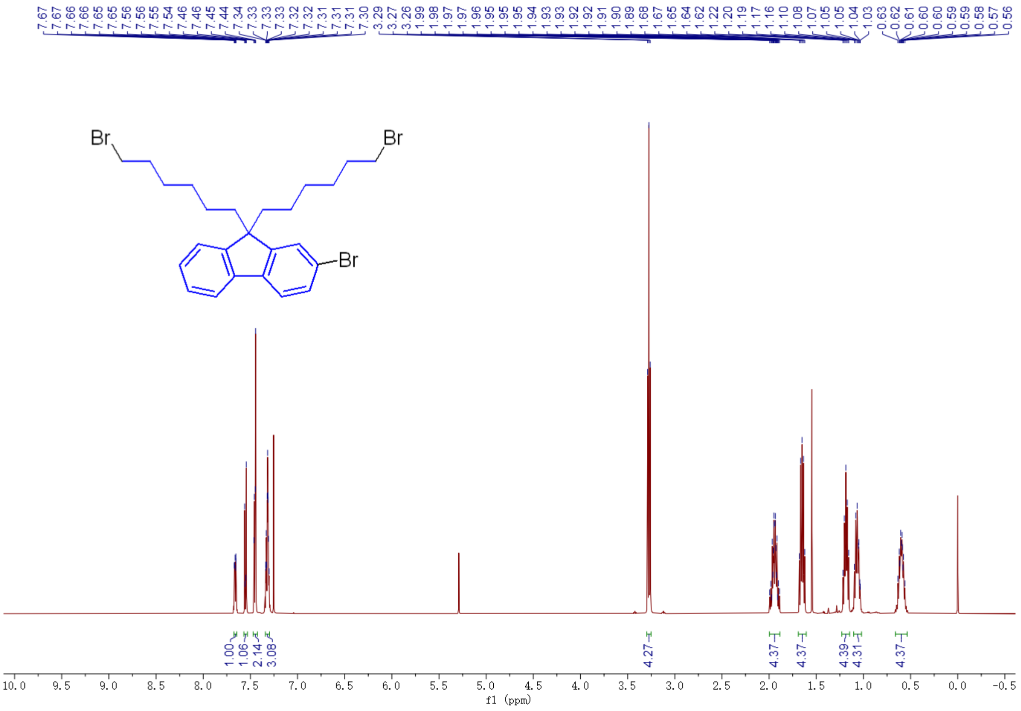

Fig. S2 ^{1}H NMR of compound **2**.

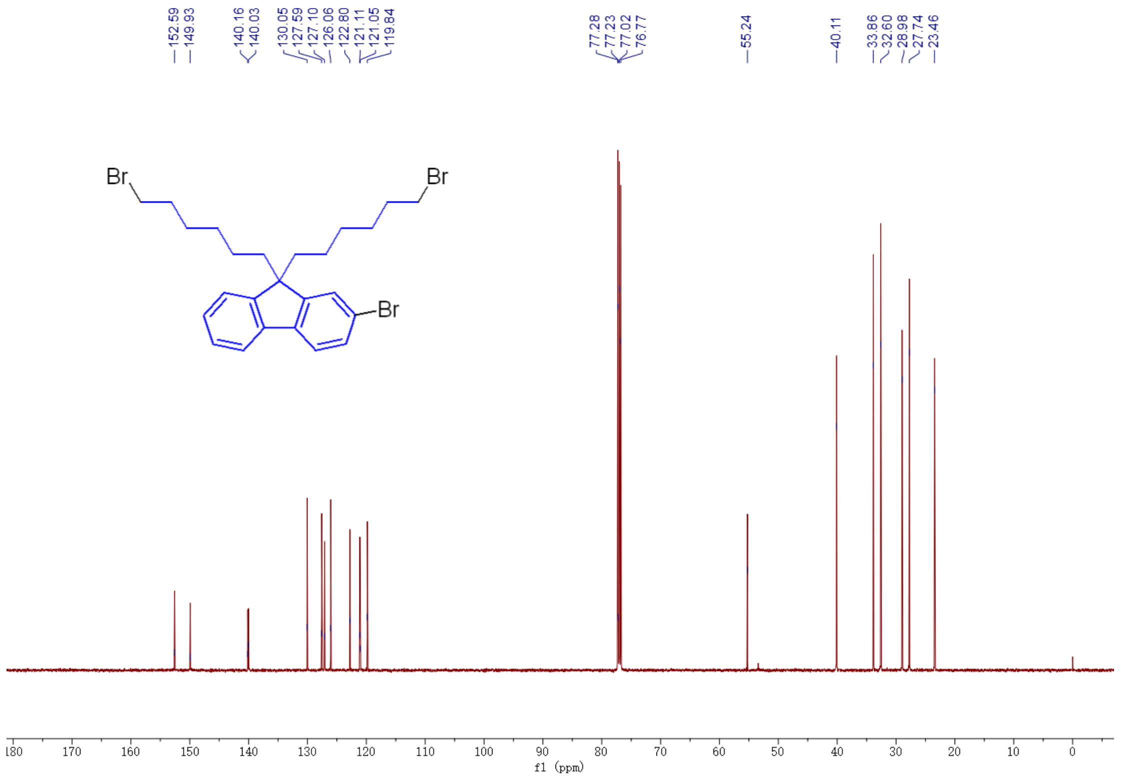

Fig. S3 ^{13}C NMR of compound **2**.

Tributyl(2,3-dihydrothieno[3,4-*b*][1,4]dioxin-5-yl)stannane (4): For the solution of compound **3** (2.13 g, 15.00 mmol) in THF (25.00 ml) under nitrogen (- 78 °C), *n*-BuLi solution (2.50 M in hexane, 7.20 ml, 18.00 mmol) was added dropwise. After the mixture was stirred at this temperature for another 2 h, tributyltin chloride (5.86 g, 18.00 mmol) was added to the solution. The reaction mixture was then slowly warmed to room temperature and stirred for overnight. After that the mixture was poured into water and extracted twice with ethyl acetate, the combined organic phase was dried with $MgSO_4$ and evaporated in vacuo without further purification.

5-(9,9-bis(6-bromohexyl)-9H-fluoren-2-yl)-2,3-dihydrothieno[3,4-*b*][1,4]dioxine (5): To a solution of compound **2** (5.71 g, 10.00 mmol) and the crude product from previous step in xylene (100 ml) under nitrogen, $Pd(PPh_3)_4$ (1.16 g) was added. The mixture was

stirred at 130 °C for 3 h. After cooling to room temperature, the mixture was poured into water and extracted twice with ethyl acetate, dried with $MgSO_4$ and evaporated in vacuo. The crude product was subjected to column chromatography on silica gel to afford IR-FE as a dark green solid (3.71 g, 58.6%). ^{1}H NMR (500 MHz, Chloroform-*d*) δ (ppm): 8.06 (s, 1H), 7.72 (dd, *J* = 8.0, 1.6 Hz, 1H), 7.69 – 7.65 (m, 2H), 7.63 (d, *J* = 1.3 Hz, 1H), 7.35 – 7.28 (m, 3H), 6.31 (s, 1H), 4.38 – 4.33 (m, 2H), 4.29 – 4.25 (m, 2H), 4.17 (t, *J* = 6.6 Hz, 2H), 3.41 (t, *J* = 6.8 Hz, 2H), 3.26 (t, *J* = 6.8 Hz, 4H), 2.01 – 1.95 (m, 4H), 1.91 – 1.83 (m, 2H), 1.71 – 1.60 (m, 6H), 1.52 – 1.44 (m, 2H), 1.44 – 1.38 (m, 2H), 1.22 – 1.15 (m, 4H), 1.11 – 1.02 (m, 4H), 0.71 – 0.60 (m, 4H). ^{13}C NMR (125 MHz, $CDCl_3$) δ (ppm): 161.13, 150.76, 150.56, 142.39, 140.87, 139.69, 138.05, 132.09, 127.00, 126.91, 124.99, 122.75, 120.26, 119.84, 119.67, 118.22, 97.31, 64.86, 64.50, 63.80, 54.99, 40.15, 33.96, 33.60, 32.62, 32.57, 29.02, 28.34, 27.71, 25.06, 23.50.

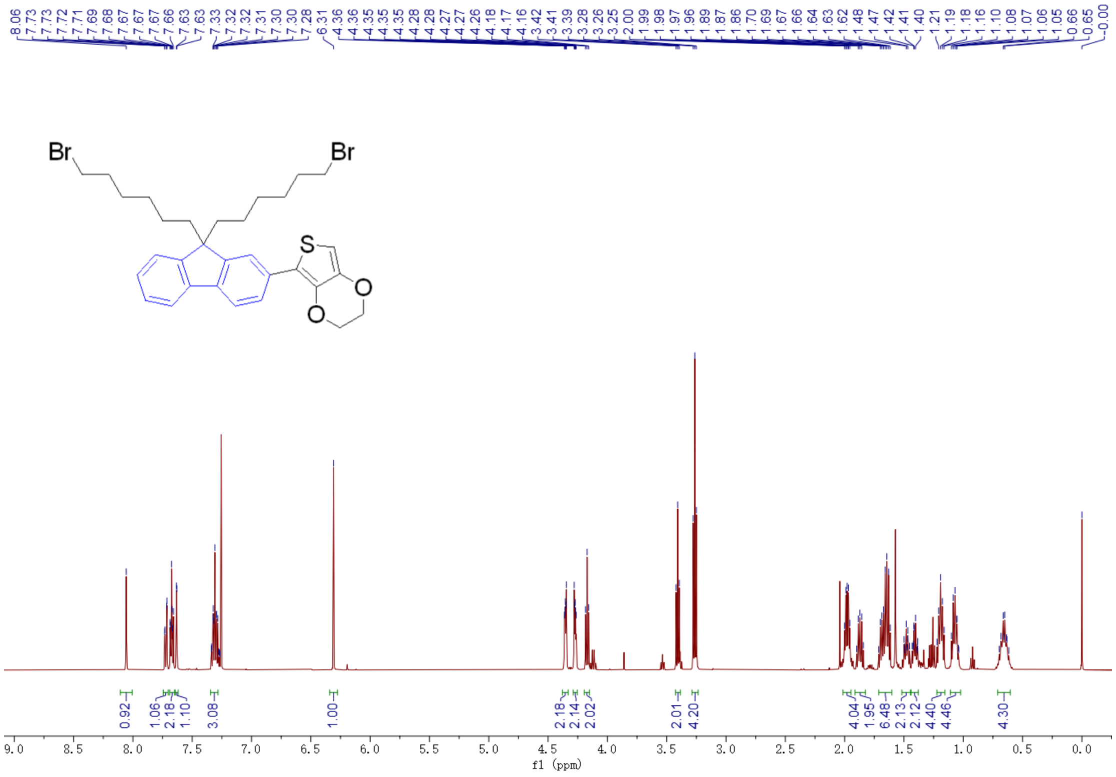

Fig. S4 ^{1}H NMR of compound **5**.

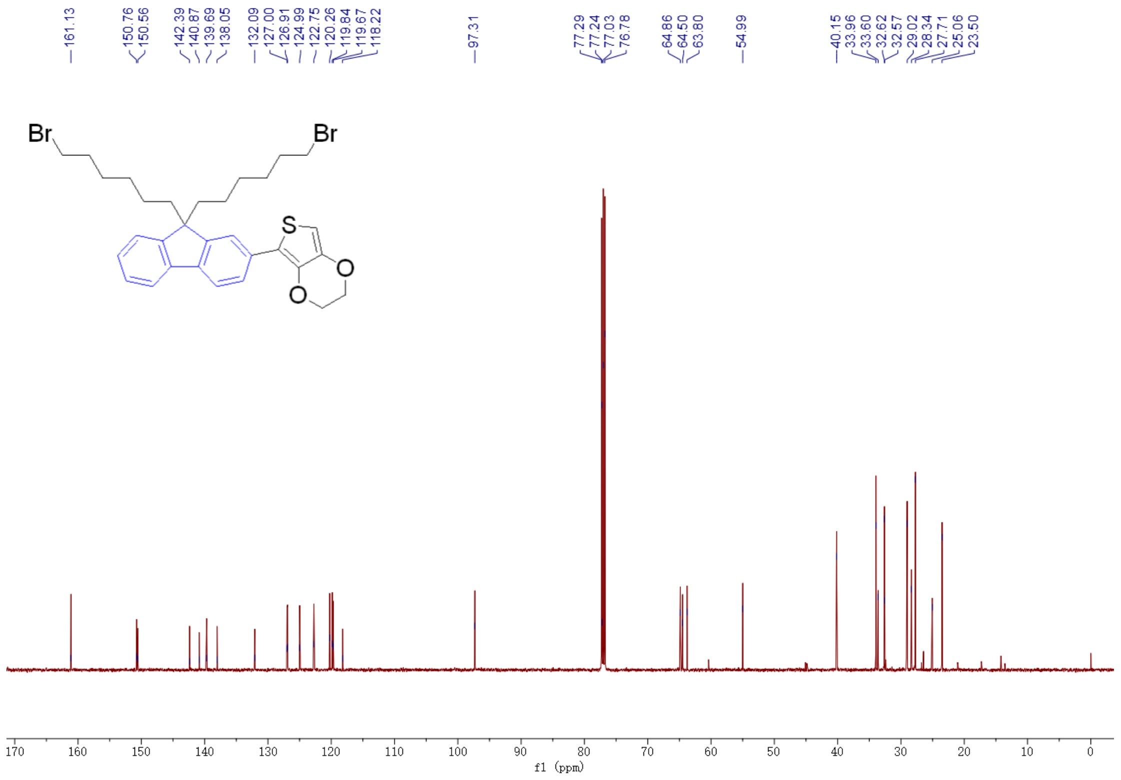

Fig. S5 ^{13}C NMR of compound **5**.

(7-(9,9-bis(6-bromohexyl)-9H-fluoren-2-yl)-2,3-dihydrothieno[3,4-*b*][1,4]dioxin-5-yl)tributylstannane (6): To a solution of compound **5** (1.90 g, 3.00 mmol) in THF (15.00 ml) at - 78 °C under nitrogen, *n*-BuLi solution (2.50 M in hexane, 1.80 ml, 4.50 mmol) was added dropwise. After the mixture was stirred at this temperature for another 2 h, tributyltin chloride (1.46 g, 4.50 mmol) was added to the solution. The reaction mixture was then slowly warmed to room temperature and stirred for overnight. After that the mixture was poured into water and extracted twice with ethyl acetate, the combined organic phase was dried with $MgSO_4$ and evaporated in vacuo without further purification.

IR-FE (7): To a solution of compound **BBTD** (352 mg, 1.00 mmol) and the crude product from previous step in xylene (20 ml) under nitrogen, Pd $(PPh_3)_2Cl_2$ (70 mg) was added. The mixture was stirred at 130 °C for 10 h. After cooling to room temperature, the mixture

was poured into water and extracted twice with ethyl acetate, dried with $MgSO_4$ and evaporated in vacuo. The crude product was subjected to column chromatography on silica gel to afford IR-FE as a dark green solid (452 mg, 31.1%). ^{1}H NMR (500 MHz, Chloroform-*d*) δ (ppm): 7.91 (dd, *J* = 7.9, 1.6 Hz, 2H), 7.77 – 7.70 (m, 6H), 7.36 – 7.30 (m, 6H), 4.55 – 4.49 (m, 4H), 4.41 – 4.36 (m, 4H), 3.28 (t, *J* = 6.8 Hz, 8H), 2.06 – 1.97 (m, 8H), 1.69 – 1.63 (m, 8H), 1.24 – 1.17 (m, 8H), 1.14 – 1.02 (m, 9H), 0.73 – 0.62 (m, 8H). ^{13}C NMR (125 MHz, $CDCl_3$) δ (ppm): 152.63, 150.81, 150.67, 141.97, 140.84, 140.37, 138.98, 138.36, 131.77, 127.19, 126.97, 125.63, 122.77, 122.69, 120.70, 119.92, 119.80, 114.03, 113.16, 108.80, 64.76, 64.60, 55.11, 40.24, 34.00, 32.64, 29.04, 27.77, 26.78, 23.55, 17.31, 13.59.

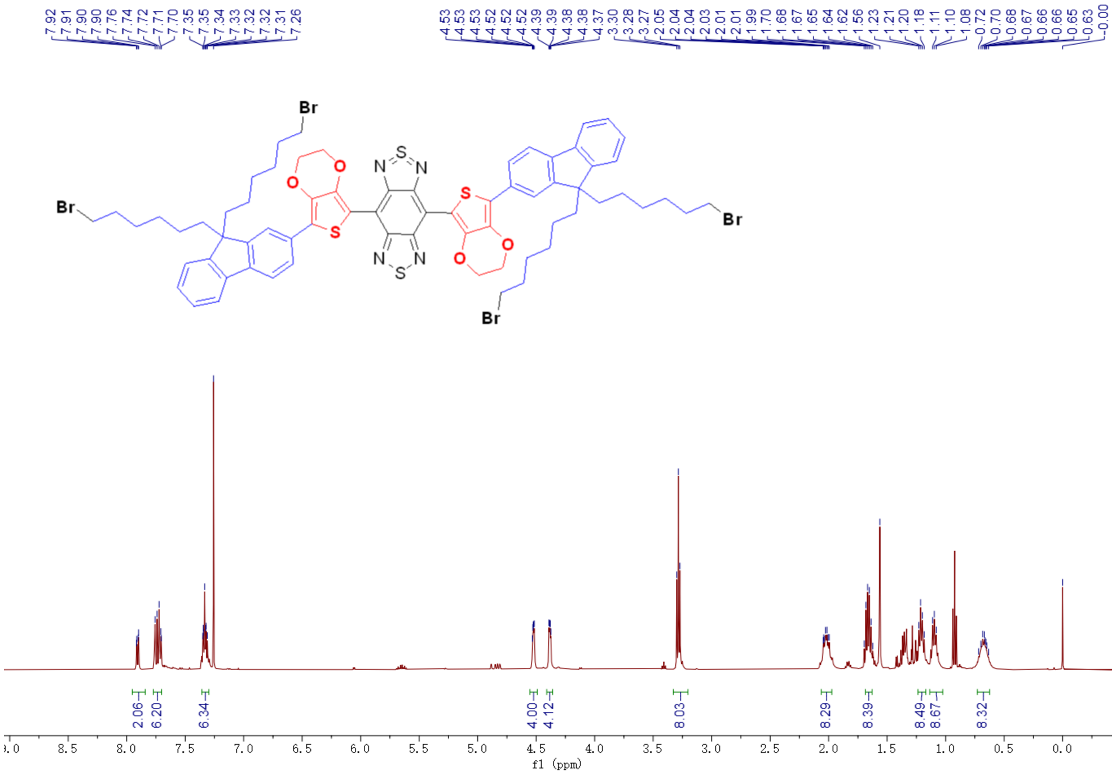

Fig. S6 ^{1}H NMR of compound **IR-FE**.

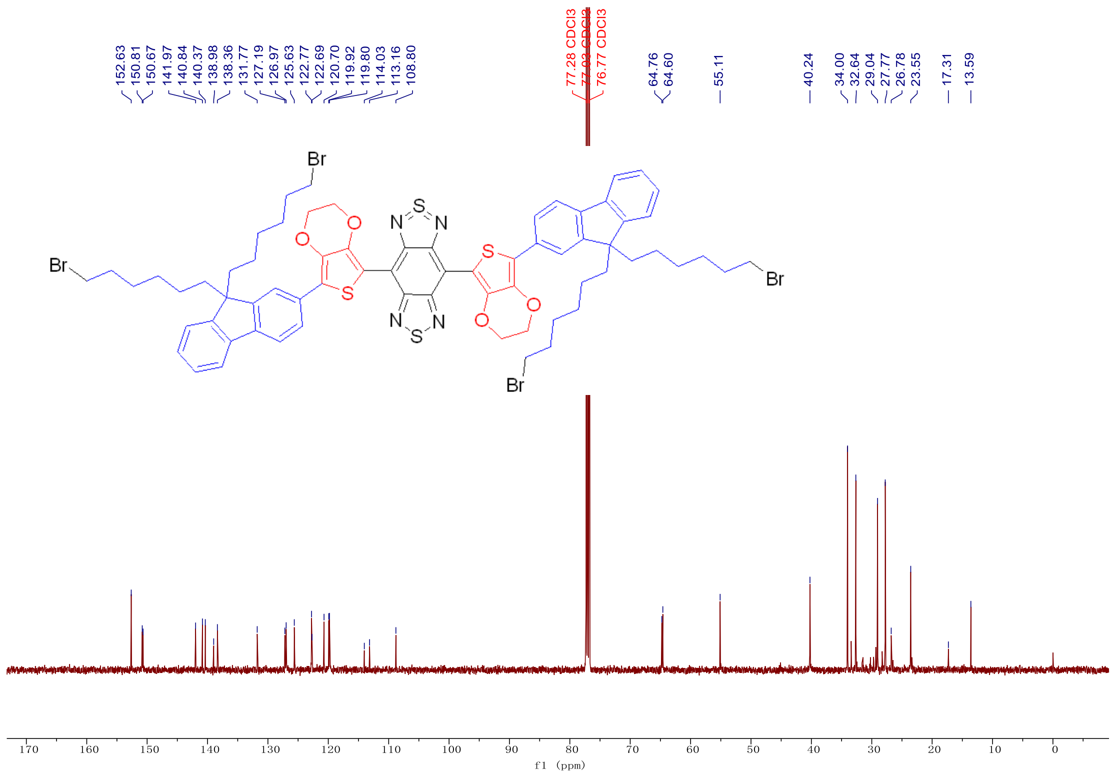

Fig. S7 ^{13}C NMR of compound **IR-FE**.

IRFE-N3 (8): Compound **IR-FE** (50 mg, 0.069 mmol) and sodium azide (47 mg, 0.72 mmol) were dissolved in DMF (3 ml) and heated for 5 h at 70 °C. After cool to room temperature, a large amount of water was added and stirred until all solids dissolved. The solution was extracted twice with ethyl acetate. The combined organic phase was washed with saturated aqueous $NaHCO_3$ solution, brine and water, then was dried with $MgSO_4$ and evaporated in vacuo. The crude product was subjected to flash column chromatography (PE/EA = 2) on silica gel to afford dark green solid (45 mg). ^1H NMR (500 MHz, Chloroform-*d*) δ (ppm): 7.90 (d, *J* = 1.7 Hz, 2H), 7.77 – 7.70 (m, 6H), 7.37 – 7.30 (m, 6H), 4.55 – 4.48 (m, 4H), 4.41 – 4.35 (m, 4H), 3.13 (t, *J* = 7.0 Hz, 8H), 2.06 – 1.98 (m, 8H), 1.45 – 1.37 (m, 8H), 1.20 – 1.06 (m, 16H), 0.74 – 0.61 (m, 8H). ^{13}C NMR (125 MHz, $CDCl_3$) δ (ppm): 152.66, 150.83, 150.68, 142.00, 140.87, 140.40, 138.39, 131.79, 127.21, 127.00, 125.66, 122.78, 122.71, 120.70, 119.94, 119.82, 114.04, 108.80, 64.76, 64.61,

55.13, 51.41, 40.26, 31.45, 30.22, 29.44, 28.68, 26.32, 23.60.

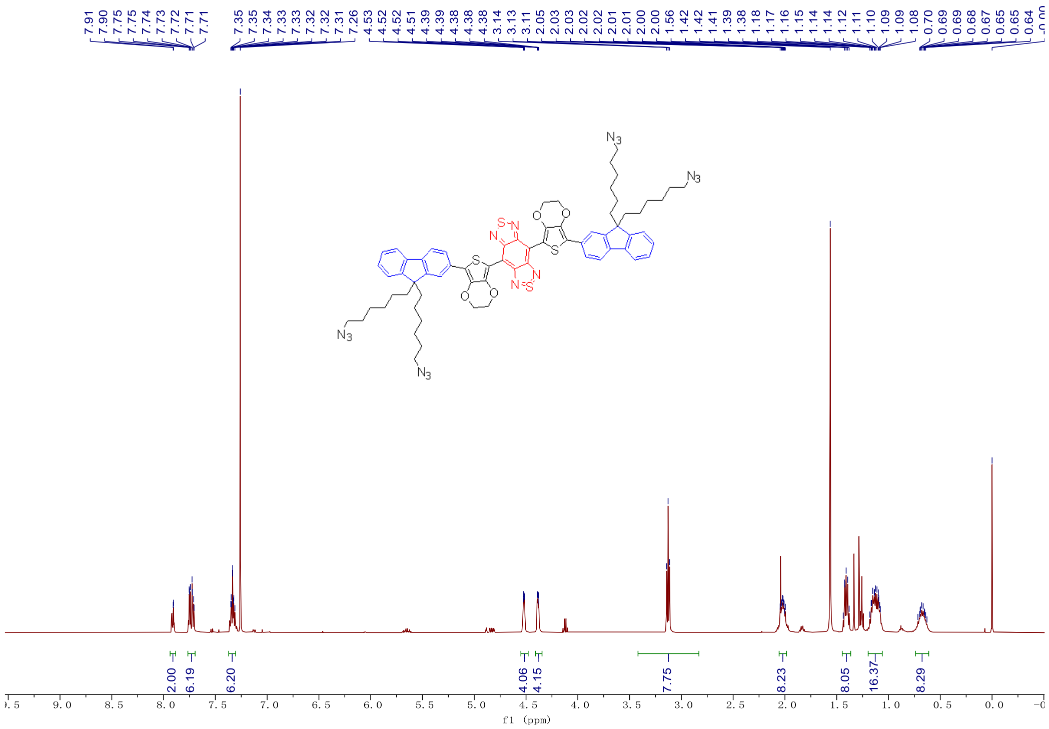

Fig. S8 ^{1}H NMR of compound **8**.

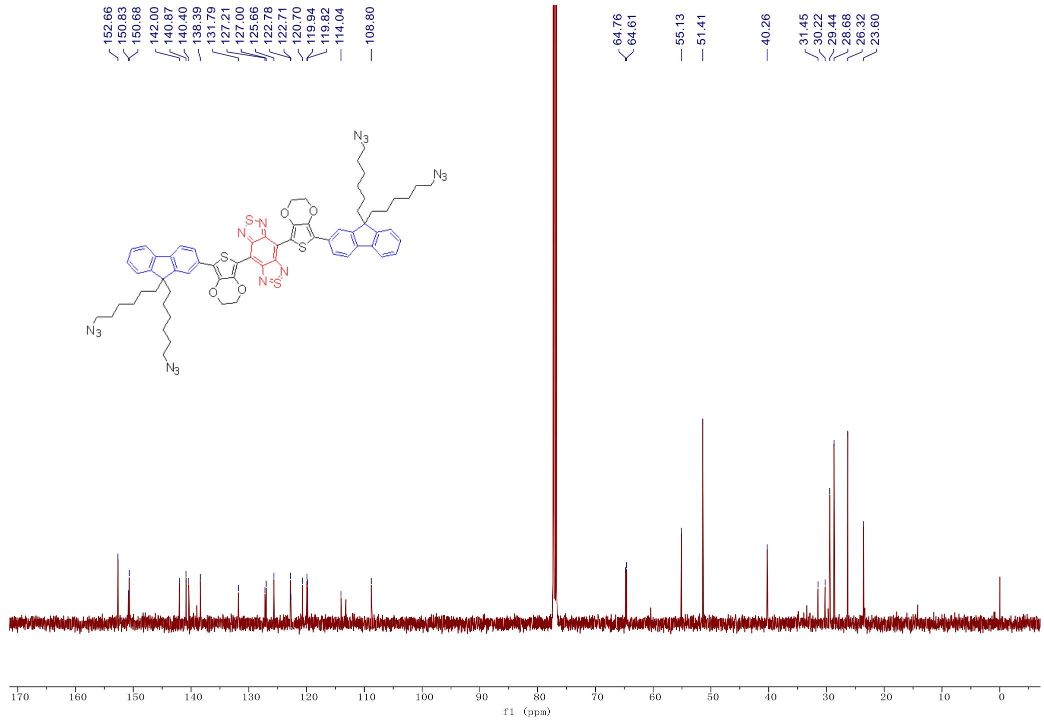

Fig. S9 ^{13}C NMR of compound **8**.

FEPT (**10**): The compound **8** (50 mg) was dissolved in 5 ml THF and copper(I) thiophene-2-carboxylate (CuTc) (10 mg), w-alkynyl-PEG- triphenylphosphonium bromide (*Mw* = 2000) (192 mg), and tris[(1-benzyl-1H-1,2,3-triazol-4-yl)methyl]amine (TBTA) (5 mg) were added. The system was stirred at RT for 2 h, then filtered with diatomite. The solvent was evaporated *in vacuo*. Then the crude product was purified by column chromatography under DCM/MeOH = 7:1 to give 177 mg of green viscous oil **10** (**FEPT**) in 87% yield. ^{1}H NMR (500 MHz, Chloroform-*d*) δ (ppm): δ 8.01 – 7.65 (m, 40H), 7.38 – 7.31 (m, 6H), 4.58 – 4.49 (m, 4H), 4.42 – 4.37 (m, 4H), 4.24 (t, *J* = 4.7 Hz, 8H), 4.21 – 4.15 (m, 6H), 4.10 (t, *J* = 4.9 Hz, 4H), 4.01 – 3.96 (m, 8H), 3.80 – 3.50 (m, 740H), 2.40 – 2.36 (m, 8H), 2.04 – 1.95 (m, 8H), 1.12 – 1.04 (m, 16H), 0.70 – 0.57 (m, 8H) ^{13}C NMR (125 MHz, $CDCl_3$) δ (ppm): 156.80, 156.05, 152.57, 140.79, 135.44, 135.42, 133.68, 133.60, 130.76, 130.66, 117.92, 117.24, 113.95, 108.67, 80.27, 71.69, 70.68, 70.60, 70.54, 69.46, 69.15, 64.60, 64.46, 64.29, 64.15, 55.13, 40.99, 35.23, 31.86, 30.84, 30.05, 29.70, 29.57, 29.52, 29.32, 29.29, 29.25, 27.37, 23.73, 23.34, 22.64, 14.12, 13.76, 8.67. ^{31}P NMR (500 MHz, Chloroform-*d*) δ (ppm): 20.47. HRMS (ESI) m/z [2654.4934±22(m+n)] [$(M-2Br)^{2+}$].

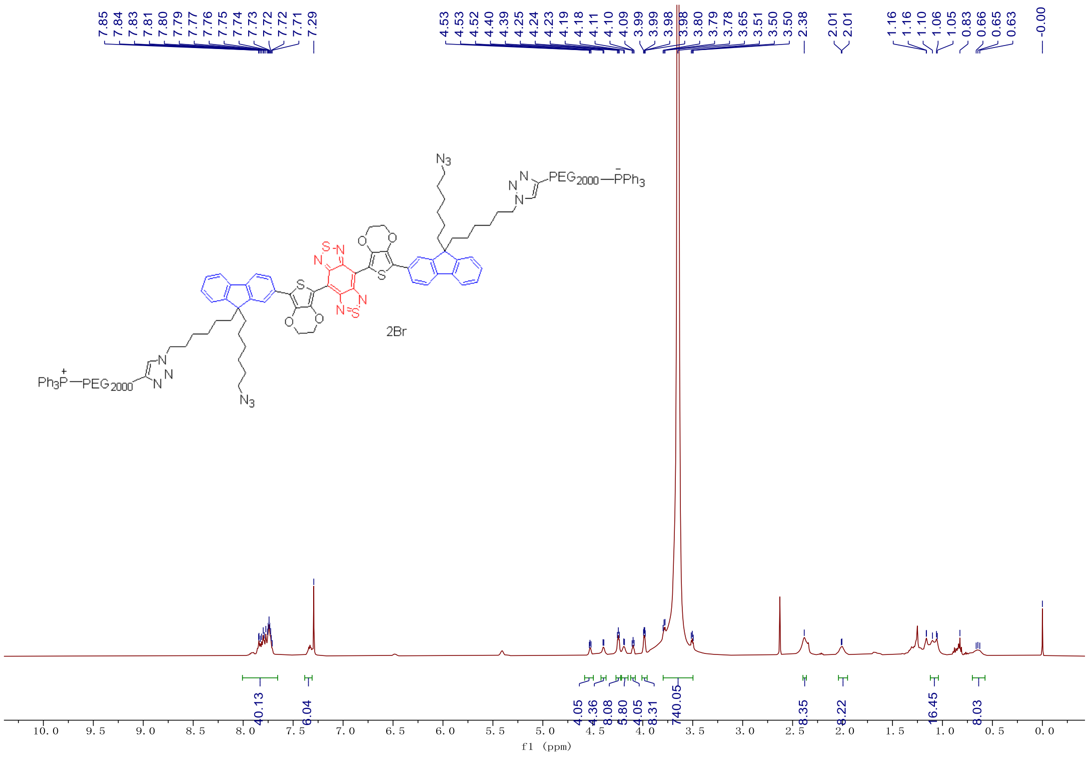

Fig. S10 ^{1}H NMR of compound **FEPT**.

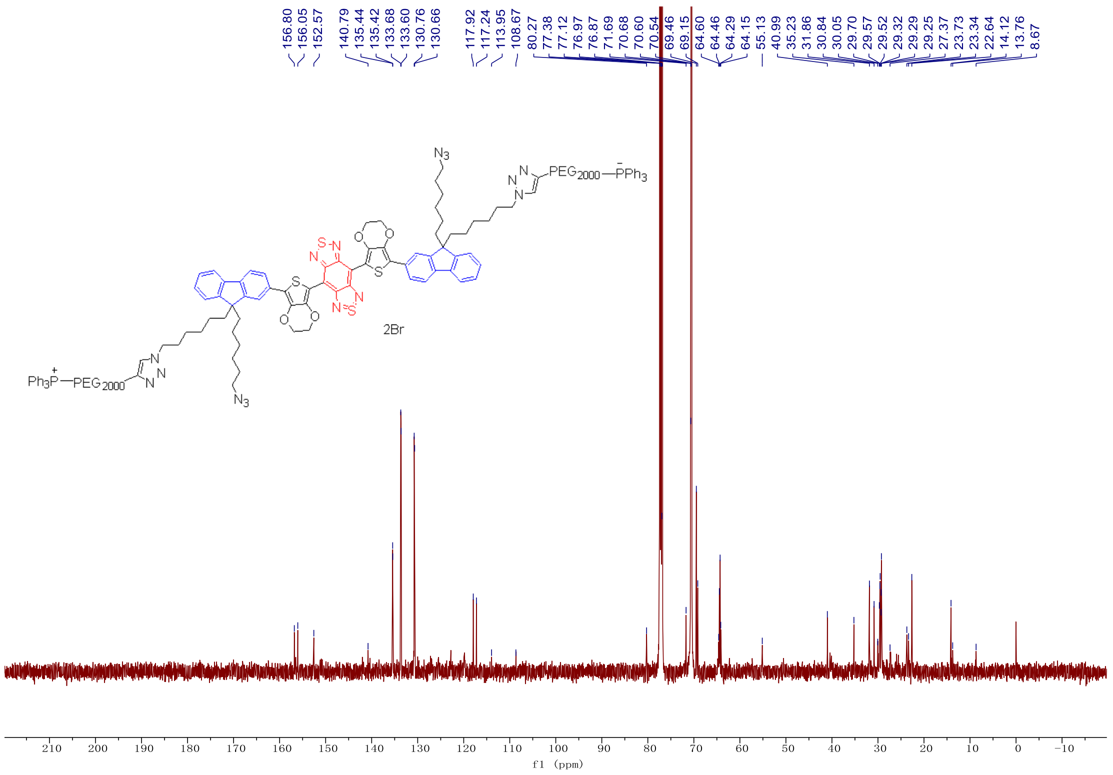

Fig. S11 ^{13}C NMR of compound **FEPT**.

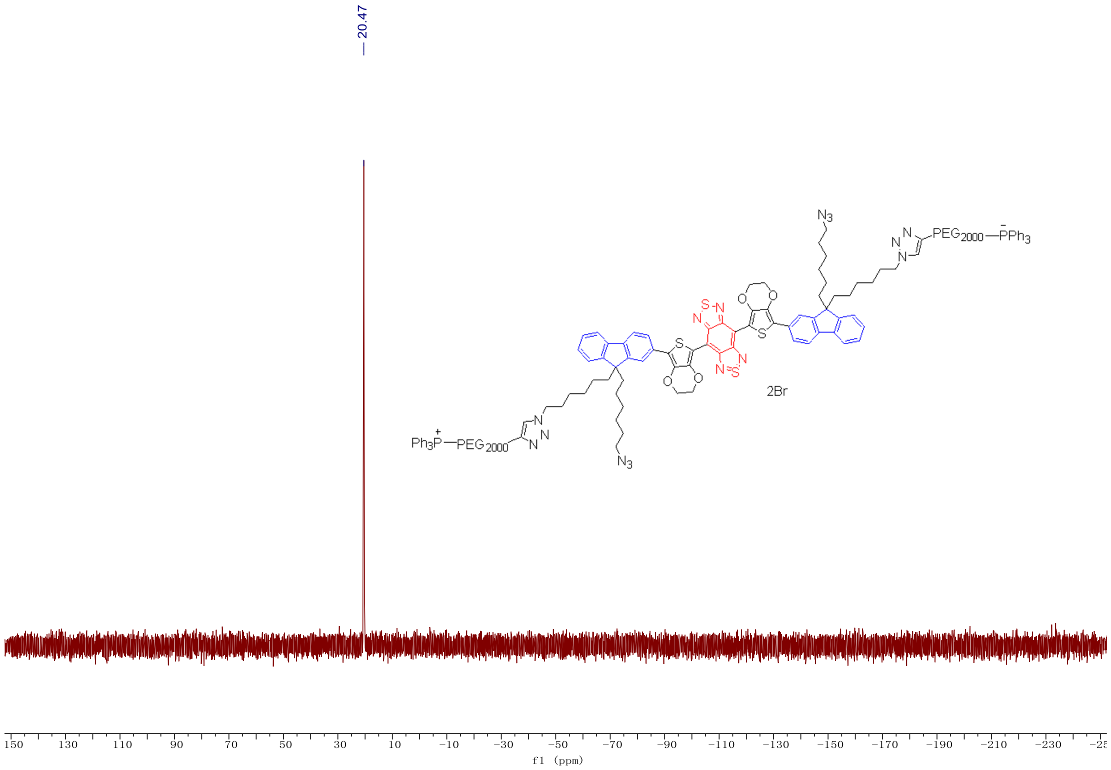

Fig. S12 ^{13}P NMR of compound **FEPT**.

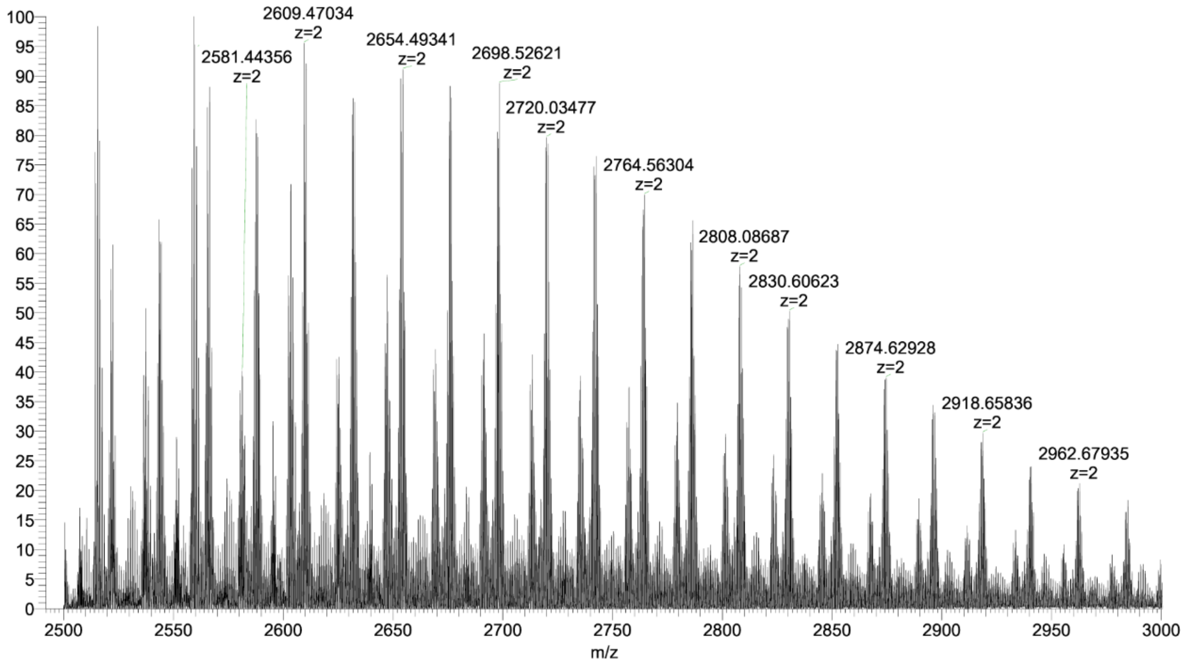

Fig. S13 HRMS of compound **FEPT.**

FEP (**11**): The compound **8** (20 mg) was dissolved in 5 ml THF and copper(I) thiophene-2-carboxylate (CuTc) (5 mg), w-alkynyl-PEG (*Mw* ≈ 2000) (38 mg), and tris[(1-benzyl-1H-1,2,3-triazol-4-yl)methyl]amine (TBTA) (1 mg) were added. The system was stirred at RT for 2 h, then filtered with diatomite. The solvent was evaporated *in vacuo*. Then the crude product was purified by column chromatography under DCM/MeOH = 7:1 to give 39 mg of green viscous oil **10** (**FEP**) in 90% yield. ^{1}H NMR (500 MHz, Methanol-d_4) δ 8.03 – 7.56 (m, 10H), 7.29 (s, 6H), 4.58 – 4.41 (m, 8H), 4.38 – 4.24 (m, 3H), 4.23 – 4.03 (m, 8H), 3.64 – 3.55 (m, 622H), 2.73 – 2.55 (m, 185H), 2.04 – 1.76 (m, 8H), 1.59 (d, J = 70.1 Hz, 8H), 0.94 – 0.80 (m, 16H), 0.53 (s, 8H). ^{13}C NMR (125 MHz, Methanol-d_4) δ (ppm): δ 151.98, 144.44, 142.44, 140.71, 138.85, 132.06, 123.59, 120.11, 109.34, 79.49, 78.44, 74.93, 71.60, 70.18, 69.99, 69.38, 68.75, 63.68, 61.92, 57.81, 57.68, 54.97, 49.80, 48.55, 39.13, 31.71, 29.79, 29.44, 29.39, 29.33, 29.29, 29.11, 28.92, 27.88, 26.60, 25.83, 22.40, 18.89, 13.38, 13.24, 12.83. HRMS (ESI) m/z [4149.4325±44(m+n)] $^{+}$].

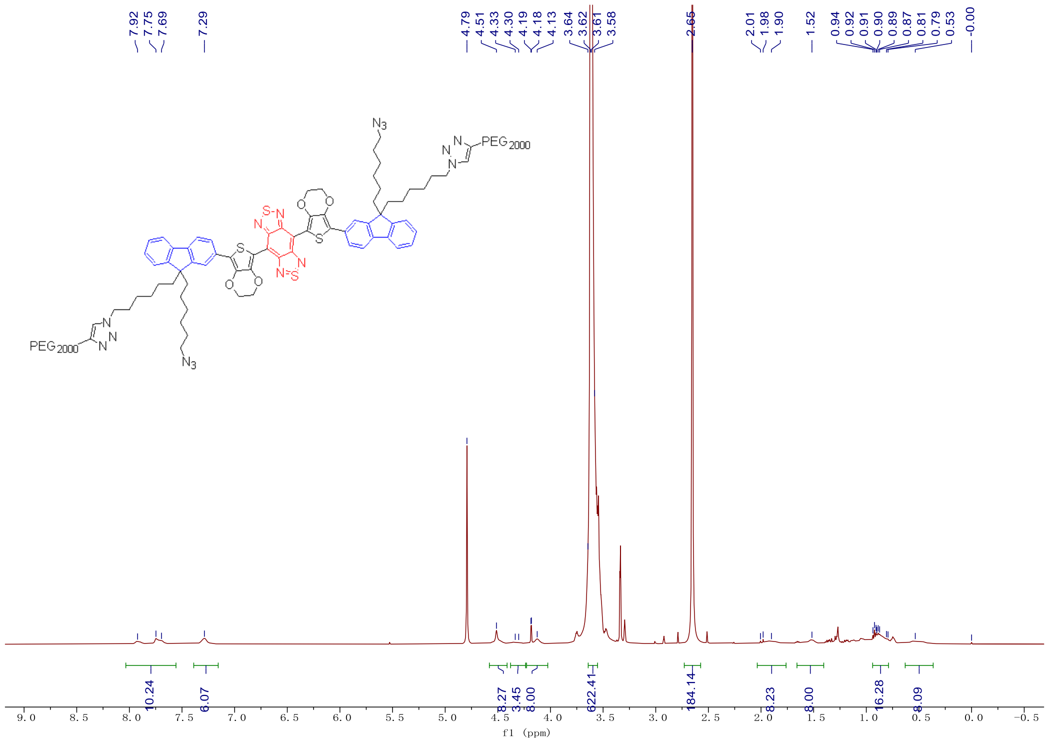

Fig. S14 ^{1}H NMR of compound **FEP**.

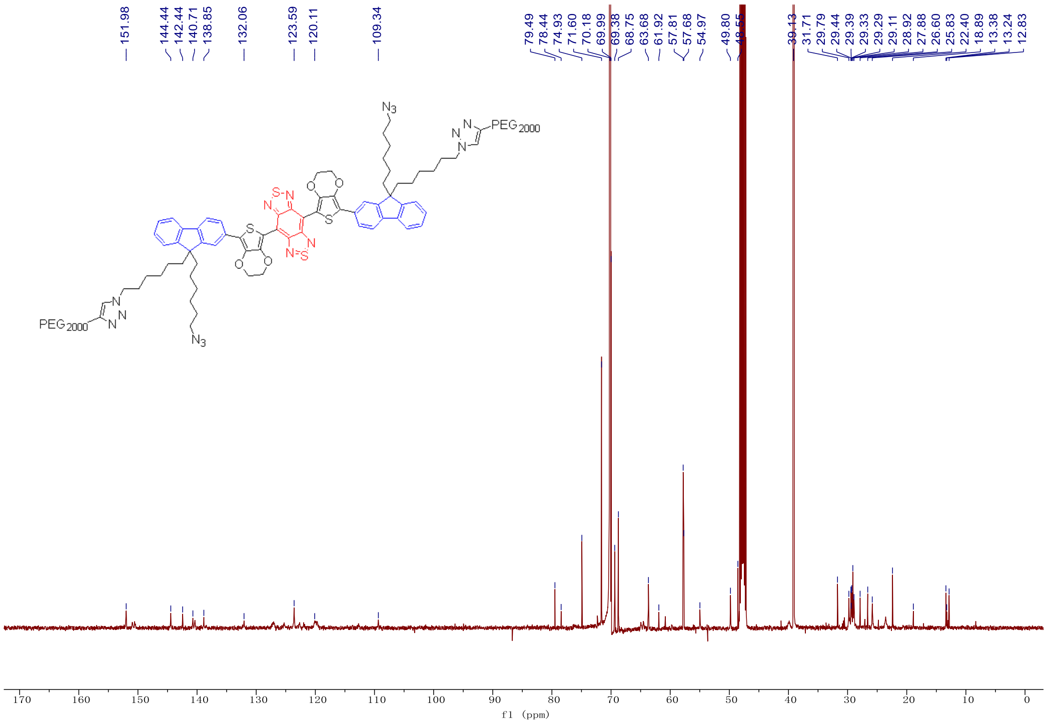

Fig. S15 ^{13}C NMR of compound **FEP**.

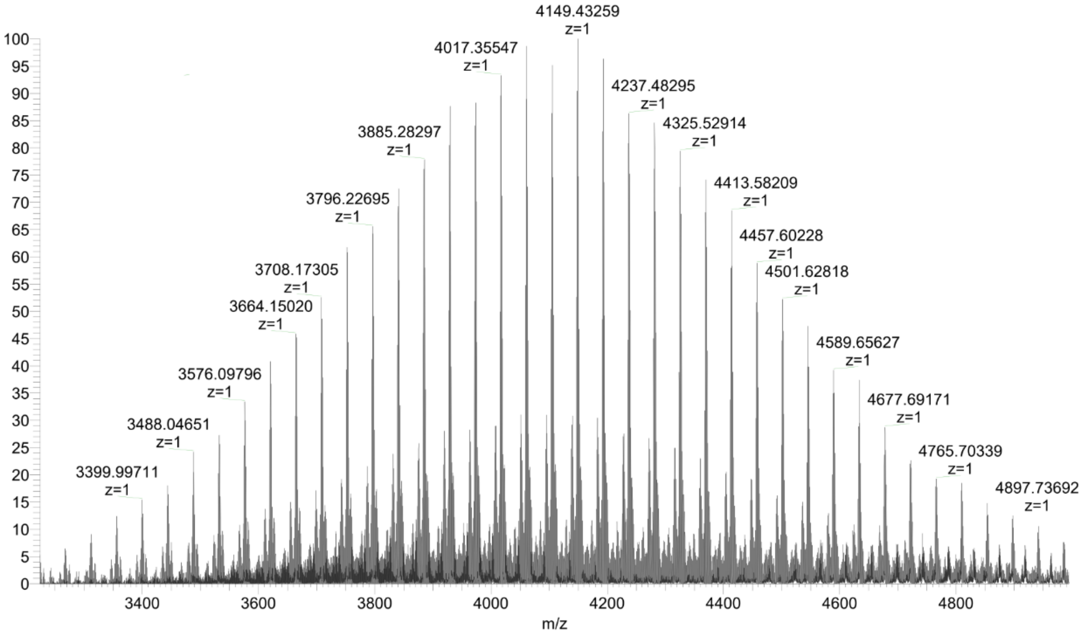

Fig. S16 HRMS of compound **FEP**.

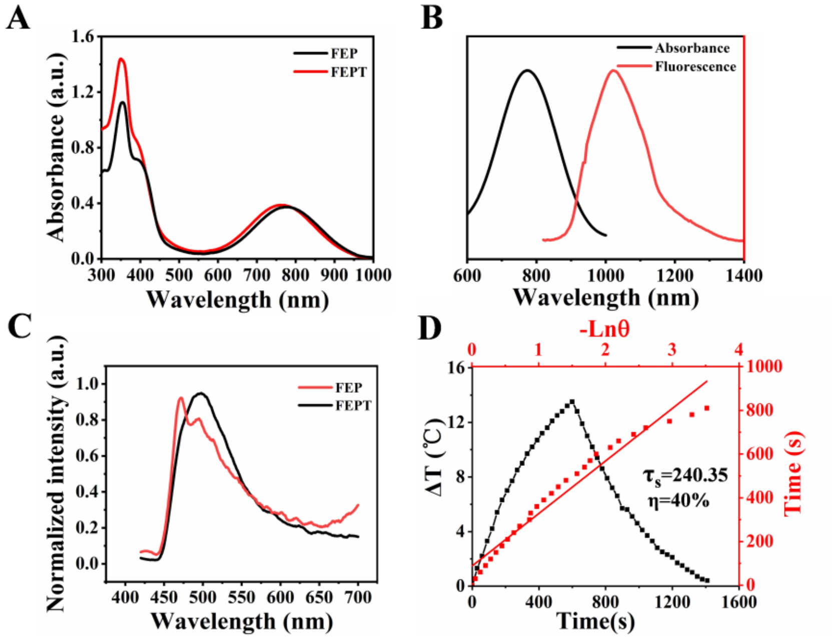

Fig. S17 A UV-Vis absorbance spectra of FEP and FEPT. **B** UV-Vis absorbance of FEP (black line) and NIR-II fluorescence emission of FEP (red line) under 808 nm laser excitation (solvents: water). **C** The fluorescence emission of FEP (red line) and FEPT (black line) under 405 nm laser excitation (solvents: water). **D** Photothermal conversion performance curves of FEP.

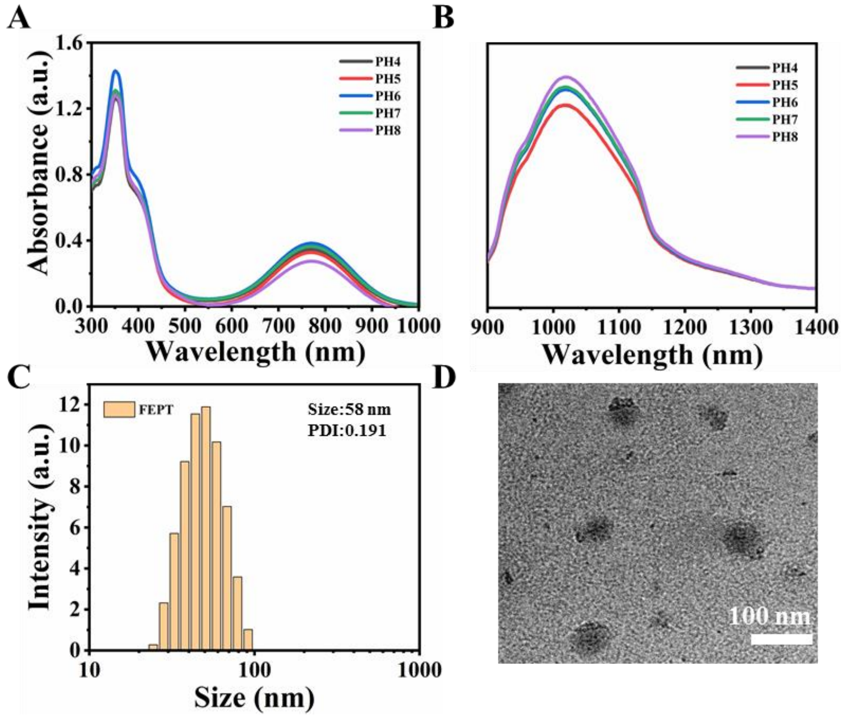

Fig. S18 A UV-Vis absorbance stability of FEPT in different PH solutions. **B** NIR-II fluorescence stability of FEPT with a peak at 1085 nm under 808 nm laser excitation (solvents: different PH solutions). **C** The size of FEPT measured by dynamic light scattering (solvents: water). **D** The TEM of FEPT in water. Scale bar:100 nm

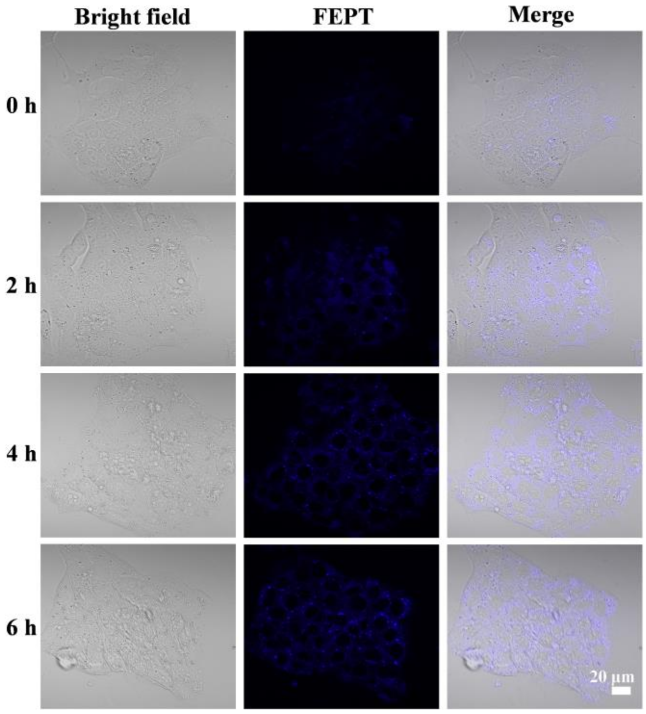

Fig. S19 Cellular uptake images of FEPT in 4T1 cells captured by LSM980. Scale bar: 20 μm.

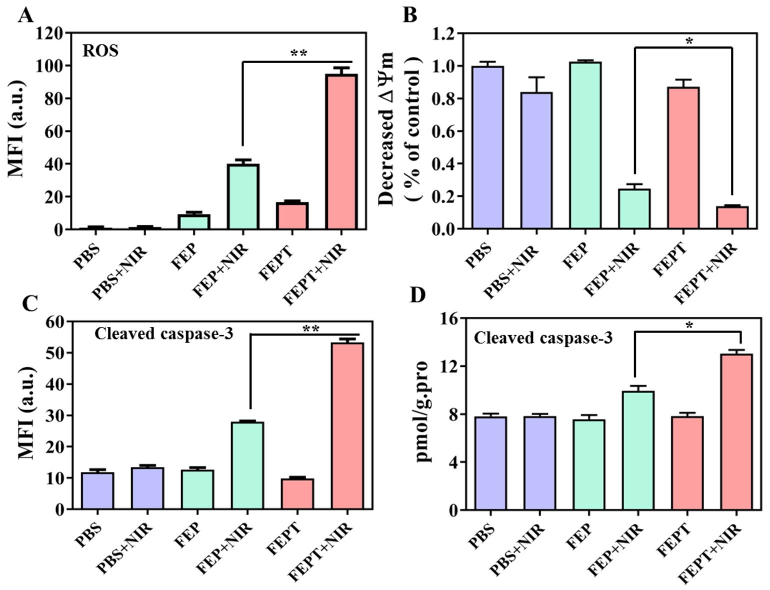

Fig. S20 A Mean fluorescence intensity of ROS in 4T1 cells detected with DCFH-DA probe after different treatments. **B** Changes in mitochondrial membrane potential (ΔΨm) of the relative fluorescent intensity of JC-1 aggregates and monomer in 4T1 cells with different treatments. **C** Quantitative fluorescence intensity of cleaved caspase-3 in 4T1 cells with different treatments analyzed by CLSM. **D** Cleaved caspase-3 detection of 4T1 cells after different treatments by ELISA. Error bars: mean ± SD (n = 3). *P < 0.05; **P < 0.01; ***P < 0.001.

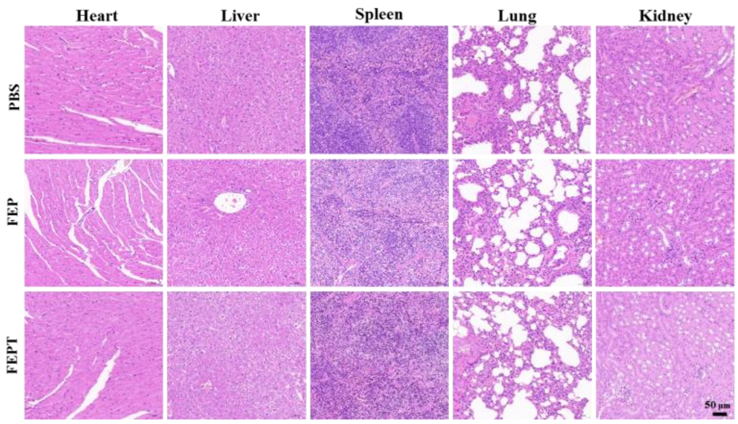

Fig. S21 H&E staining images of five major organs (heart, liver, spleen, lung and kidney) in the healthy Balb/c mice after intravenous injection of PBS, FEP and FEPT without 808 nm laser irradiation at 15 d post-injection. Scale bar: 50 μm.

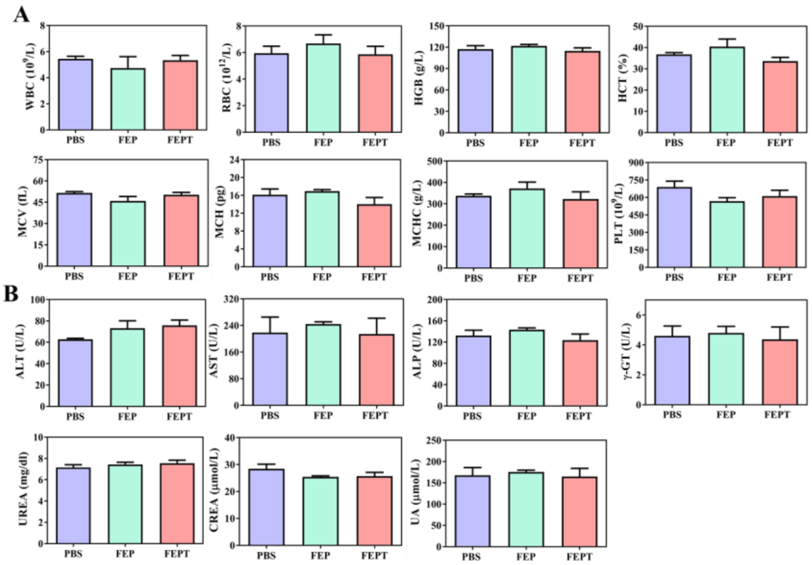

Fig. S22 A Blood routine examinations in the PBS, FEP and FEPT groups. **B** Chemistry indexes of the liver and renal function tests in the PBS, FEP and FEPT groups. All the tests are detected in the healthy Balb/c mice without NIR irradiation at 15 d post-injection.

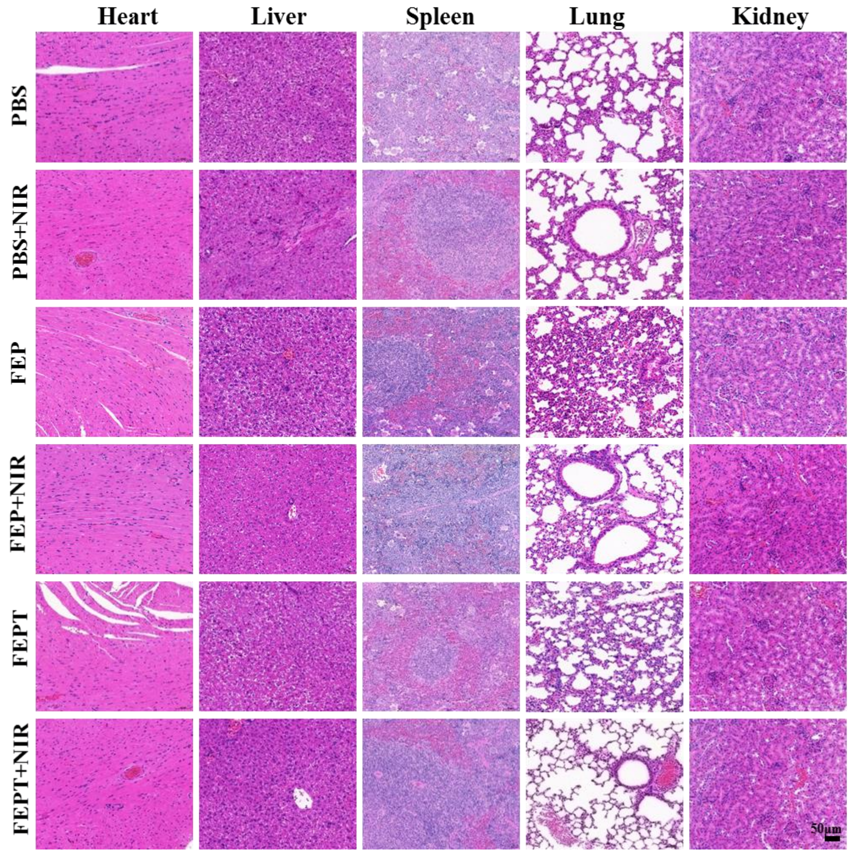

Fig. S23 H&E staining images of five major organs (heart, liver, spleen, lung, and kidneys) in 4T1 tumor-bearing mice with different treatments at 15 d post-injection. Scale bar: 50μm.

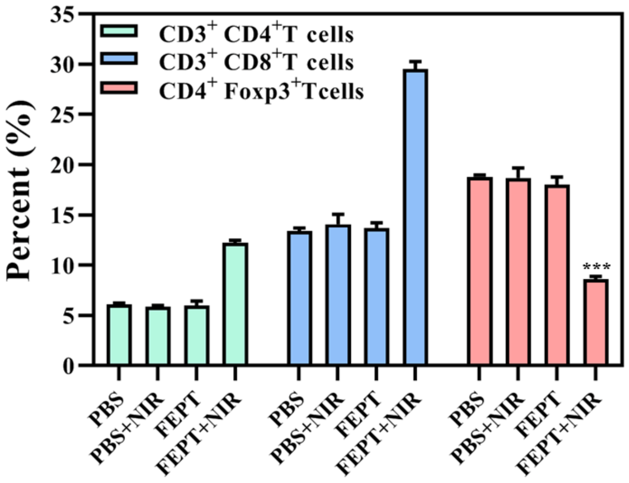

Fig. S24 Quantitative results of $CD3^+CD4^+T$ cells, $CD3^+CD8^+T$ cells, and Tregs in $CD4^+T$ cells in splenic lymphocytes isolated from different-treated mice. Mean ± SD (n = 3). *P < 0.05; **P < 0.01; ***P < 0.001.

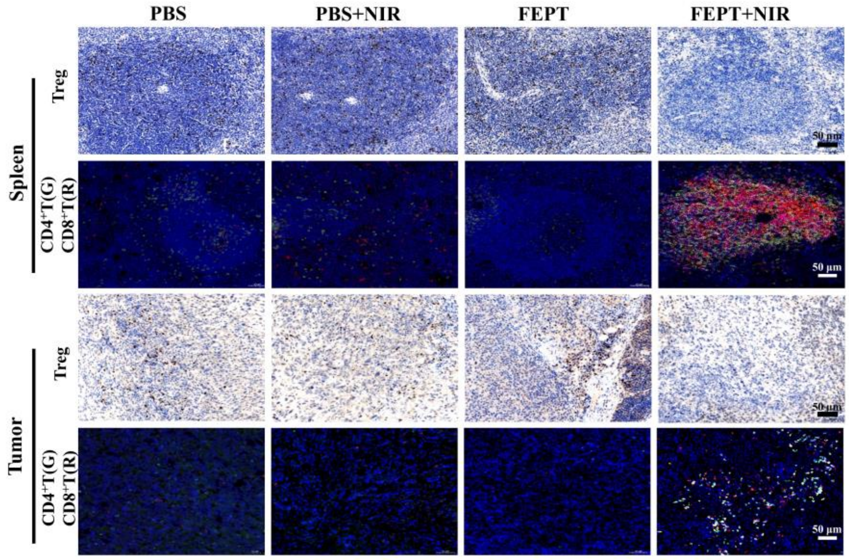

Fig. S25 The IHC analysis of Tregs and the IF analysis of infiltrating $CD4^+T$ (green) and $CD8^+T$ (red) cells in the spleen and tumor tissues. The blue signals in IHC and IF staining indicate cellular nuclei that stained with hematoxylin and Hoechst 33258, respectively. The positive signal of Tregs in IHC staining is brown. Green and red fluorescence signal indicates $CD4^+T$, $CD8^+T$ cells, respectively. Scale bar: 50 μm.

Reference

1. Diao S, Hong G, Robinson JT, Jiao L, Antaris AL, Wu JZ, Choi CL, Dai H: Chirality Enriched (12,1) and (11,3) Single-Walled Carbon Nanotubes for Biological Imaging. *J Am Chem Soc*

2012, 134:16971-16974.
2. Yang Q, Ma Z, Wang H, Zhou B, Zhu S, Zhong Y, Wang J, Wan H, Antaris A, Ma R, et al: Rational Design of Molecular Fluorophores for Biological Imaging in the NIR-II Window. *Adv Mater* 2017, 29:1605497.
3. Wang Q, Xu J, Geng R, Cai J, Li J, Xie C, Tang W, Shen Q, Huang W, Fan Q: High performance one-for-all phototheranostics: NIR-II fluorescence imaging guided mitochondria-targeting phototherapy with a single-dose injection and 808 nm laser irradiation. *Biomaterials* 2020, 231:119671.
